# Spatial and Temporal Microbial Patterns in a Tropical Macrotidal Estuary Subject to Urbanization

**DOI:** 10.3389/fmicb.2017.01313

**Published:** 2017-07-13

**Authors:** Mirjam Kaestli, Anna Skillington, Karen Kennedy, Matthew Majid, David Williams, Keith McGuinness, Niels Munksgaard, Karen Gibb

**Affiliations:** ^1^Research Institute for the Environment and Livelihoods, Charles Darwin University Darwin, NT, Australia; ^2^Power and Water Corporation Darwin, NT, Australia; ^3^Aquatic Health Unit, Department of Environment and Natural Resources, Northern Territory Government Darwin, NT, Australia; ^4^Australian Institute of Marine Science Darwin, NT, Australia

**Keywords:** temporal and spatial patterns, microbiota, macrotidal tropical estuary, treated sewage effluent, urban runoff

## Abstract

Darwin Harbour in northern Australia is an estuary in the wet-dry tropics subject to increasing urbanization with localized water quality degradation due to increased nutrient loads from urban runoff and treated sewage effluent. Tropical estuaries are poorly studied compared to temperate systems and little is known about the microbial community-level response to nutrients. We aimed to examine the spatial and temporal patterns of the bacterial community and its association with abiotic factors. Since Darwin Harbour is macrotidal with strong seasonal patterns and mixing, we sought to determine if a human impact signal was discernible in the microbiota despite the strong hydrodynamic forces. Adopting a single impact–double reference design, we investigated the bacterial community using next-generation sequencing of the 16S rRNA gene from water and sediment from reference creeks and creeks affected by effluent and urban runoff. Samples were collected over two years during neap and spring tides, in the dry and wet seasons. Temporal drivers, namely seasons and tides had the strongest relationship to the water microbiota, reflecting the macrotidal nature of the estuary and its location in the wet-dry tropics. The neap-tide water microbiota provided the clearest spatial resolution while the sediment microbiota reflected current and past water conditions. Differences in patterns of the microbiota between different parts of the harbor reflected the harbor's complex hydrodynamics and bathymetry. Despite these variations, a microbial signature was discernible relating to specific effluent sources and urban runoff, and the composite of nutrient levels accounted for the major part of the explained variation in the microbiota followed by salinity. Our results confirm an overall good water quality but they also reflect the extent of some hypereutrophic areas. Our results show that the microbiota is a sensitive indicator to assess ecosystem health even in this dynamic and complex ecosystem.

## Introduction

Estuarine ecosystems are a focal point of impacts from the land and seaward side and experience increasing pressures from growing populations and human activities worldwide (Jennerjahn and Mitchell, 2013). Within these ecosystems, tidal creeks are especially dynamic environments renowned for their complexity and productivity (Holland et al., 2004). They can show signs of impairment years before deeper open estuarine habitats, and may provide early warning of ecological and public health threats (DiDonato et al., 2009). The headwater regions of urbanized creeks are often the first indicators of water quality degradation and creek sediments are repositories for much of the pollution released into the environment.

The bacterial community has been shown to be a sensitive indicator of ecosystem stress in estuaries modified by anthropogenic disturbance in temperate Australia (Sun et al., 2012; Jeffries et al., 2016). In contrast to temperate systems, knowledge on drivers of the bacterial community in tropical macrotidal estuaries is sparse. The few reports available for tropical systems have shown that sediment microbial communities were spatially and temporally dependent and associated with changes in temperature, nutrient load, dissolved oxygen, salinity and pH (Sun et al., 2011; Zhang et al., 2014). Hydrological and sedimentological processes of macrotidal estuaries with a tidal range of more than 4 m are mainly controlled by semidiurnal and fortnightly tidal cycles with large variations in mixing, and ratios of freshwater to marine water inputs (Allen et al., 1980). One study in a macrotidal estuary in the Australian wet-dry tropics found that salinity was the determining factor for temporal variations of nitrogen-cycle related bacteria in the sediment (Abell et al., 2009).

Darwin Harbour, in the wet-dry tropics of Northern Australia, is an estuarine ecosystem subject to increasing human pressure. While two thirds of its catchment is still undeveloped, the harbor is subject to considerable on- and off-shore infrastructure and population growth with over 130,000 people living in its watershed (Aquatic Health Unit, 2016). It is a macrotidal environment and, as a consequence, pollutants are commonly assumed to disperse rapidly (Burford et al., 2008). Accordingly, it is still considered to be in a relatively pristine condition and nitrogen-limited with the extensive area of fringing mangroves responsible for the bulk of primary production (Burford et al., 2008; Butler et al., 2013; Aquatic Health Unit, 2015). However, some areas of the harbor have been shown to be poorly flushed and have a complex bathymetry that can trap pollutants inshore for long periods (Williams et al., 2006). Treated sewage effluent discharged from four wastewater treatment outfalls has been identified as the dominant anthropogenic point-source of nutrients to the harbor. Effluent has been found historically to contribute 71% of total phosphorus and 31% of total nitrogen of the annual catchment load entering the harbor (Skinner et al., 2009). In comparison, diffuse urban runoff based on 2004 land-use categorization, was estimated to contribute 16% of total phosphorus and 21% of total nitrogen (Skinner et al., 2009) and a hydrodynamic model for Darwin Harbour raised concerns about the increasing significance of nutrient and pollutant inputs from diffuse urban sources in particular during the wet season (Drewry et al., 2009).

Considering the increasing human pressure, there is a need to develop tools that assess the ecosystem health of the harbor. Currently, there are no systematic microbiology data for Darwin Harbour despite the fact that microbes drive many of the biogeochemical processes in mangrove sediment and algal dynamics in tidal creeks affected by effluent (Nogales et al., 2011; Reed and Martiny, 2013). Filling this knowledge gap would provide important new information of relevance to macrotidal tropical harbors world-wide.

The objective of this study was to describe the spatial and temporal patterns of the bacterial community and abiotic factors associated with the microbiota in water and sediment across Darwin Harbour during the dry and wet season of the Indo-Australian monsoon and during neap and spring tide cycles. During spring tides tidal currents peak at 2 m s^−1^ in Darwin Harbour and the tidal range reaches up to 7.4 m which compares to just 1.5–3 m for neap tides (Burford et al., 2008). We predicted strong season- and tide-related microbial patterns with considerable mixing of fresh- and sea-water characteristic of dynamic estuarine macrotidal ecosystems (Meire et al., 2005). We tested the hypothesis that even with the strong hydrodynamic forces present an effluent specific bacterial signal would be discernible in water or sediment.

## Materials and methods

### Study area

Darwin Harbour is lined by mangroves, with extensive intertidal mudflats, and subject to a tropical savannah climate, with a distinct dry and wet season and average annual rainfall of 1,727 mm (www.bom.gov.au). The harbor is adjacent to the city of Darwin, which has a population growth rate of 1.9%; the second fastest of all Australian capital cities in 2014–2015 (www.abs.gov.au).

**Figure 1:**
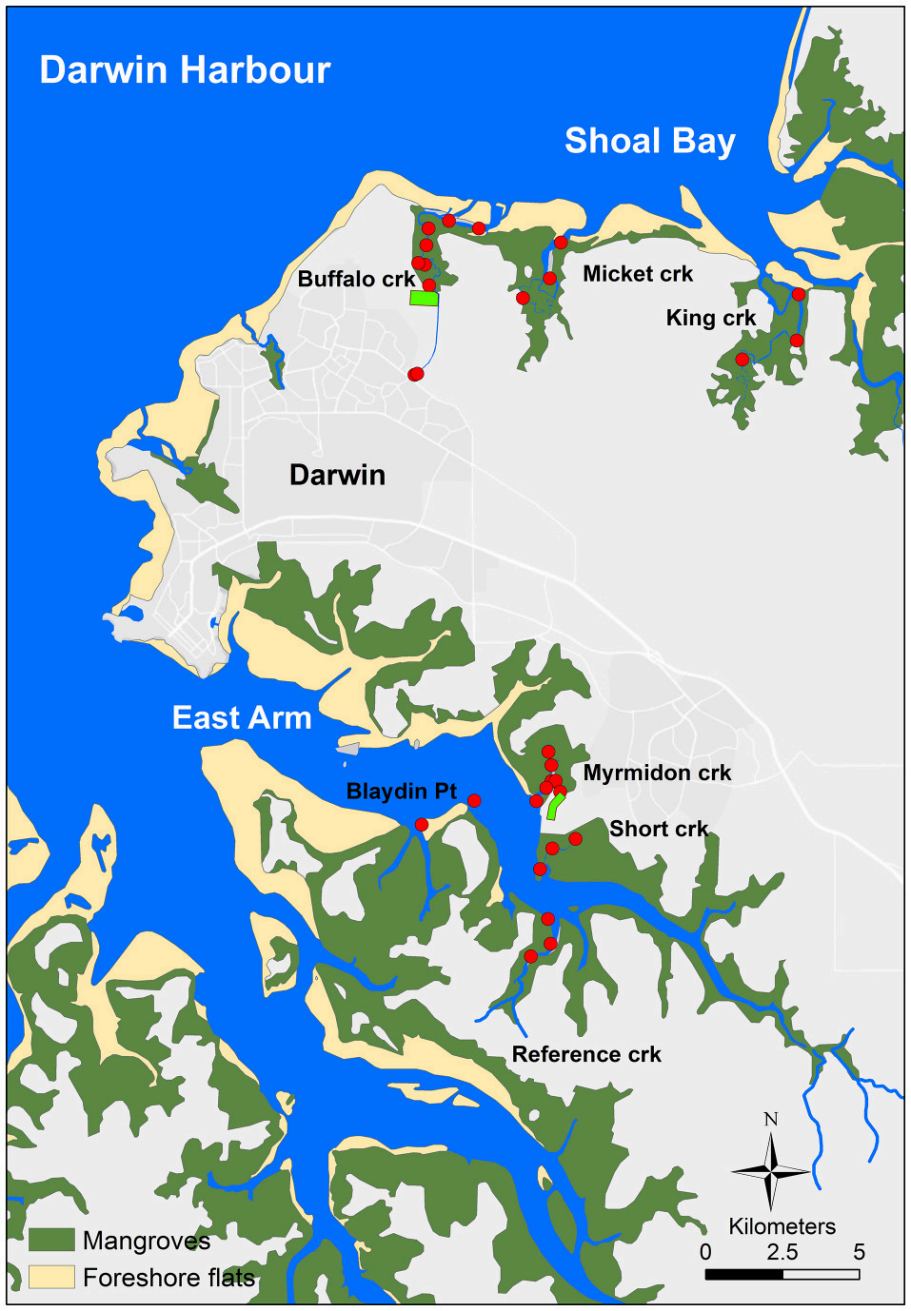
The Darwin Harbour map. Darwin Harbour in northern Australia with sampling sites in Shoal Bay and East Arm. The light green rectangles at Buffalo and Myrmidon Creek indicate the wastewater treatment ponds.

Buffalo Creek, a tidal creek in Shoal Bay in the north of the harbor (Figure 1) receives secondary-treated sewage effluent (140 *t* total *N* year^−1^ and 26 *t* total *P* year^−1^ in 2013) from an effluent outfall in the headwaters of the creek, 4.8 km from its mouth. The estimated population of the serviced area of the waste stabilization ponds that discharge to Buffalo Creek was 47,466 in 2013. The ponds consist of two parallel treatment trains with five waste stabilization ponds in each series (one facultative and four maturation) designed to remove organic matter, nutrients and fecal bacteria and viruses. Hypereutrophic Buffalo Creek has a narrow meandering channel through mangroves with multiple barriers to direct water exchange and tidal movement including a large barrier sand bar across the mouth (Figure 1). Suburban stormwater and runoff also flow into the headwaters of Buffalo Creek and parts of it were also artificially filled for mosquito control. Micket and King Creeks are the reference creeks for Shoal Bay (Figure 1) with less human impact although Micket Creek still receives some urban runoff. Micket Creek also has a sandbar at the mouth albeit smaller. There are extensive salt flats behind the fringing mangroves of the Shoal Bay creeks.

In the East Arm area of the harbor (Figure 1), Myrmidon Creek receives secondary-treated sewage effluent (109 *t* total *N* year^−1^ and 18 *t* total *P* year^−1^ in 2013) from an outfall for five waste stabilization ponds in series (one facultative and four maturation), servicing the city of Palmerston with estimated 31,216 households in 2013. The effluent is discharged into a 600 m long tributary which branches off the main creek 900 m upstream from its mouth. Myrmidon Creek has a wide and relatively straight channel surrounded by mangroves and is considered oligotrophic to locally mesotrophic. Reference Creek and Short Creek are the reference creeks for East Arm, as well as two sites off Blaydin Point in the middle of East Arm (Figure 1). Both, Myrmidon and Short Creek also receive urban stormwater.

### Sites and sampling regime

Water and sediment were collected from 32 sites in the Shoal Bay and East Arm areas in Darwin Harbour (Figure 1). For Shoal Bay, seven sites were chosen along the impacted Buffalo Creek between the site where the effluent entered the creek channel through a pipe and the sandbank at the mouth. A site at the headwaters of Buffalo Creek close to Darwin suburbs was included to represent tidal creek water mixed with urban runoff (Figure 1). Treated sewage effluent and urban runoff were also collected before mixing with the creek water. There were three sites each from two reference creeks, Micket and King Creeks. The distance between the mouth to the effluent outfall or most upstream site for the reference creeks was similar i.e., 4.6–4.8 km for all three creeks. Similarly for East Arm, there were seven sites along the impacted creek (Myrmidon Creek) as well as three sites each from two reference creeks (Short and Reference Creeks). Pure effluent was collected as well as water at the outfall site where the effluent discharged across a bank into the mangroves and a Myrmidon side-tributary (Figure 1). A further site was chosen in this tributary 400 m downstream from the outfall and 200 m upstream from the Myrmidon main channel. Two sites off Blaydin Point captured the water from the center of the East Arm estuary.

There were eight rounds of sampling over 2 years with two rounds each in April 2013, Sept 2013, Feb 2014, and Sept 2014. This covered two consecutive wet and dry seasons and for water samples, a neap and spring tide sampling round for each of the seasons. For access reasons, water samples were collected just after high tide at outgoing tide and thus, represented the best-case scenario in terms of dilution of nutrient inputs from effluent or land runoff. Samples were collected from a depth of 0.5 m and in duplicates from key sites during neap tide. It was only possible to collect sediment during neap tides. Using a corer the top 10 cm were collected in duplicates. All samples were kept on ice and processed within 6 h of collection. Water samples were aliquoted and parts filtered *in situ* using 0.45 μm inline filters for subsequent nutrients analysis while water for subsequent molecular analysis was filtered in the laboratory within 6 h of collection (see below) and filters were frozen at −20°C. Sediment was aliquoted for molecular and physicochemical analysis and frozen (see below).

### Physicochemistry and nutrients analysis

#### Water

Water physicochemistry was measured *in situ* using a multi-probe YSI (www.YSI.com) measuring pH, conductivity, salinity, turbidity, temperature, depth and dissolved oxygen (% and mg/L) at an average depth of 0.5 m where possible (less for effluent and urban runoff). The multiprobe was calibrated according to manufacturer's specifications prior to each fieldtrip and readings of calibration solutions were checked upon return to the lab. Using flow injection analysis (FIA) at the Environmental Chemistry & Microbiology Unit (ECMU) (CDU, Darwin, Australia), PO_4_-P, total dissolved phosphorus (TDP), total dissolved nitrogen (TDN), ${\text{NH}}_{4}^{+}$-N, NO_2_-N, and NO_3_-N were measured from filtered water and total phosphorus (TP) and total nitrogen (TN) from unfiltered water. Analytical quality control included replicate analyses, analysis of spike additions and certified reference materials (GJ 1051, GJ1153, GJ1195, Graham Jackson Pty Ltd.). Chlorophyll-a concentration was also determined at ECMU by fluorometric analysis calibrated by a chlorophyll-a standard (Sigma Aldrich, Australia) and solid secondary standards (Turner Designs). Total and dissolved organic carbon were measured at the Australian Institute of Marine Science (AIMS) (Townsville, Australia) using a SHIMADZU TOC-5000A (www.shimadzu.com). Total suspended solids (TSS) were measured by filtration (0.45 μm) and weighing of filtered particulate material at ECMU for water samples of the 2nd year of the study.

#### Sediment

Using FIA, total Kjeldahl nitrogen (TKN) concentrations were measured in sediment samples at ECMU and total organic carbon concentrations in sediment were measured at AIMS (<2 mm fraction). The concentrations of Al, P, S, Ca, V, Fe, Mn, Cu, Zn, As, Cd, and Pb were measured in HNO_3_+HClO_4_ acid extracts of the <2 mm fraction by ICPMS (AGILENT 7700ce, www.agilent.com) at ECMU. Analytical quality control included replicate analyses, analysis of spike additions and certified reference materials (MESS-3, National Research Council Canada). The oxidation redox potential (ORP) of the sediment was measured *in situ* for the 2nd year of the study using an ORP meter calibrated with Zobell's solution (YSI).

### Water and sediment DNA extraction

#### Water

Within 6 h of collection, on average 500 mL of creek water, 1 L of marine water and 50 mL of effluent were filtered using sterile 0.45 μm filters (cellulose nitrate, 47 mm, Sartorius, Australia). Filters were frozen at −20°C until further processing. DNA was extracted using the PowerWater DNA Isolation kit (MoBio, Carlsbad, CA, USA) following the manufacturer's instructions.

#### Sediment

On average, 7 g of sediment were aliquoted within 6 h of collection and frozen until processed for DNA extraction using the PowerMax Soil DNA Isolation kit (MoBio, Carlsbad, CA, USA) following the manufacturer's instructions. DNA eluent in 5 mL were precipitated and eluted in 100 μL before further processing.

### Next generation sequencing

On average, 1.5 μg of dried DNA were sent to mrdnalab (www.mrdnalab.com, Shallowater, TX, USA) for 16s rRNA gene amplicon sequencing using the MiSeq platform (Illumina, San Diego, CA, USA). The 16-s rRNA gene was amplified using the V4/V5 primers 16S_F563/16 (5-AYTGGGYDTAAAGNG) and 16S_BSR926/20 (5-CCGTCAATTYYTTTRAGTTT) (Claesson et al., 2010) with barcodes on the forward primer. The HotStarTaq Plus Master Mix Kit (Qiagen, USA) was used with the following conditions: 94°C 3 min, 28 cycles of 94°C 30 s, 53°C 40 s, 72°C 1 min, final elongation step at 72°C for 5 min. Pooled PCR products were purified using calibrated Ampure XP beads and a DNA library was prepared following the Illumina TruSeq DNA library preparation protocol. Sequencing was performed on a MiSeq Illumina sequencing platform following the manufacturer's guidelines using the v2 chemistry for the first two rounds of samples and v3 for the remaining six rounds.

### Processing of sequencing data

The Usearch-8 fastq (www.drive5.com) and FastQC (www.bioinformatics.babraham.ac.uk) tools were used to assess the quality of the MiSeq R1 and R2 fastq files. Four MiSeq processing pipelines were compared; Mothur (Schloss et al., 2009), UPARSE (Edgar, 2013), QIIME closed reference and QIIME open reference (Caporaso et al., 2010). The QIIME open reference pipeline was chosen (see reasoning Figure S1, Table S1). QIIME 1.9.0 was used on a Linux cluster. In short, paired ends of R1 and R2 files were joined using the QIIME default parameters. Quality control was conducted using the Usearch 8 fastq-filter with a minimum length threshold of 330 bp (median length 372 bp) (Edgar, 2010), an expected error threshold of 1 and no N's allowed. The forward and reverse complement of R1 and R2 sequences were checked for barcodes and forward primers and the sequences were demultiplexed. The maximum unacceptable Phred quality score was set to 20. Chimeras were removed using the Usearch8 uchime_ref command and the QIIME “gold” reference database (Edgar et al., 2011). OTUs were picked using the default parameters incl. the Uclust method at 97% similarity. For taxonomy assignment, the Greengenes database was used (release May 2013) (McDonald et al., 2012). Singleton OTUs and OTUs assigned to Archaea were excluded. The sequencing depth ranged from 10,711 to 588,040 sequences per sample (median 68,842 sequences). To account for differences in filtering volume, DNA extraction and PCR efficiency and sequencing depth, all samples were subsampled to 17,000 sequences. This number was chosen as a trade-off between excluding a minimum number of samples with <17,000 sequences (10 sediment and three water samples were excluded) while still retaining an average Goods coverage of above 90% for all remaining 274 water samples. A flattening of the rarefaction curves was observed for both, water and sediment samples (Figure S2). A second stage analysis (Primer-E 7, Plymouth UK) compared the weighted UniFrac resemblance matrices of all water and sediment samples rarefied to different subsampling depths (i.e. no subsampling, subsampling to 17,000 or 20,000 sequences). The distance matrices showed high Spearman correlations of >0.99 between the different subsampling depths, indicating that the effect of subsampling upon the beta analysis was negligible for both water and sediment samples (Table S1, Figure S1).

### Data analysis

Data were analyzed using Primer-E 7 (Plymouth, UK), StataIC 14 (www.stata.com), R studio (R v3.2.2) and QIIME 1.9.1. Alpha diversity (Simpson diversity and Goods coverage) was assessed in QIIME (alpha_diversity.py) and differences tested using Kruskal-Wallis in Stata. Beta diversity was assessed using a weighted UniFrac distance matrix at OTU level based on the abundance weighted fraction of the total phylogenetic branch length not shared between a pair of samples (Lozupone et al., 2011). The weighted UniFrac distance matrices were visualized using unconstrained principal coordinates analyses (PCO).

#### Hypothesis testing

Hypothesis testing was used for Shoal Bay and East Arm separately to compare spatial (between creeks) and/or temporal (years, seasons or tides) differences. A PERMANOVA (Primer-E 7) crossed design with type III partial sums of squares was used with fixed factors tide, year of sampling, season and creek for water and sediment. A factor for site was also included, nested in creeks, to account for the repeated measures design. Sites were chosen based on distance of the site to the mouth so this factor was also fixed (Quinn and Keough, 2002). Effluent and urban runoff sites (before mixing with the creeks) were excluded as being not representative of the creeks. As an additional test, DNA concentration was added as a cofactor to the PERMANOVA with type I (sequential) sums of squares to test for technical bias with no changes to the overall results (data not shown).

#### Microbial predictive ability for location and association with abiotic factors

A canonical analysis of principal coordinates (CAP, Primer-E 7), constrained by creek, tested the predictive ability of the microbiota for sampling location. A leave-one-out allocation cross-validation was performed and the number of incorporated PCO axes was based on a maximized correct classification rate.

Abiotic factors were log transformed if positively skewed and compared between seasons, creeks and location using multiple linear regressions. A distance based linear model (DistLM) and redundancy analysis (dbRDA) in Primer-E were used to determine which normalized abiotic factors and nutrients best explained the variability in the microbial communities. Collinear variables (Pearson correlation >0.9) were excluded (EC, TP, TN, TOC, P-PO_4_). The Akaike information criterion (AIC) was used to select models with stepwise variable selection. A canonical correspondence analysis (CCA) (library vegan in R) was also performed for the Shoal Bay sediment microbiota. Both OTU and sample scores were scaled symmetrically by the square root of the CCA eigenvalues. The ordistep forward selection tool was used (Borcard et al., 2011) to obtain the most parsimonious CCA model with the least number of abiotic factors. Furthermore, in a nonmetric multidimensional scaling ordination (MDS) of the weighted OTU UniFrac distance matrix, the correlation between abiotic factors and the MDS axes was assessed using the function envfit of the library vegan. The function ordisurf was used to fit the surface of the most correlated nutrients to the MDS plot using generalized additive models and thinplate spline interpolation.

#### Spatial analysis and variation partitioning of the water microbiota

The spatial structure of the water microbiota at neap tide for Shoal Bay and East Arm was assessed using eigenvector-based spatial modeling (Borcard et al., 2011). A distance matrix based on distances across water between sites was used to obtain x and y coordinates for the sampling sites using classical (metric) multidimensional scaling (function cmdscale in library MASS in R). The Hellinger standardized OTUs of neap water microbiota were checked for linear spatial trends across the x and y coordinates in a RDA analysis (vegan). Principal Coordinates of Neighbor Matrices (PCNM library in R) was used to identify non-linear spatial trends at different scales. Orthogonal independent spatial eigenvectors for neighboring sampling sites exhibiting positive spatial correlation (based on Moran's I) were identified and their association with the microbiota tested in a RDA using forward selection. Stopping rules were applied if the adjusted R^2^ exceeded the R^2^ of the global model or if the *P* value exceeded 0.05 (Borcard et al., 2011). Variation partitioning was conducted differentiating between these spatial trends, salinity and/or nutrient levels with a combination of parameters that best explained the variance in the Shoal Bay and East Arm microbiota using the adjusted R^2^ in RDA analyses (function varpart, vegan) (Borcard et al., 2011).

#### Relatedness of the water microbiota

Relatedness of the water microbiota across Darwin Harbour was measured by calculating the weighted UniFrac distance matrix based on the median counts per OTU per site and a neighbor joining tree analysis across both sampling years and harbor areas i.e., Shoal Bay and East Arm. Bootstrap analysis was conducted on 500 rarefied trees. The tree and median bacterial taxa information at family level were combined with a geo-referenced map from Darwin Harbour in GenGIS 2.4.0. The map was georeferenced in ArcMap 10.1 (www.esri.com) using shapefiles obtained from the Northern Territory Government.

#### Indicator analysis

To find bacterial families significantly associated with a group of samples, an indicator species analysis (Dufrêne and Legendre, 1997) was conducted using the IndVal function in the labdsv library in R. This function calculates an indicator value (range 0 to 1) based on the within-family relative abundance in the groups and the relative frequency of that family across groups. Groups tested included creeks, tides and seasons. The permutation based *P-*values were corrected for multiple testing using the p.adjust function in R and the false discovery rate (FDR) (Benjamini and Hochberg, 1995). OTUs which occurred in <10 samples were excluded. Non-parametric Kruskal-Wallis testing was also conducted with *P-*values adjusted for multiple testing using the FDR method.

## Results

### Sample processing overview

During eight sampling rounds over 2 consecutive years (2013–2014), two dry and wet seasons and four neap and spring tides, 286 water and 208 sediment samples were collected from 32 sites from two areas in Darwin Harbour, namely Shoal Bay and East Arm creeks (Figure 1). Sixteen sediment (7.7%) and six water (2.0%) samples did not produce 16s rRNA gene amplicon sequences. Samples were rarefied to 17,000 sequences, which resulted in a mean Goods coverage of 90.2% for water samples (*n* = 277) and 79.2% for sediment samples (*n* = 192). For sediment samples, 197,507 OTUs were called by the pipeline with an average of 5,420 OTUs per sample [standard deviation (sd) 1,180]. Significantly fewer OTUs per sample were measured for water with an average of 2,391 OTUs (sd 937) and total 162,125 OTUs (Student's *t*-test *t* = 30.4, *P* = 0.001).

### The water microbiota

The association between the microbiota and seasons, tides, years and location for the East Arm and Shoal Bay areas were analyzed to determine whether the microbiota differed spatially namely between creeks, and/or temporally i.e., between tides, seasons or years.

### The influence of seasons and years

The dry and wet seasons had the greatest impact on the Darwin Harbour water microbiota. This was the dominant factor and accounted for more variation in the microbiota than any other tested factor, including the unexplained residual variance within groups of samples (Table 1). There was also season-specific clustering in particular for the East Arm water microbiota (Figure S3). Excluding the outfall and urban runoff-related sites, there were more bacteria of the family Chromatiaceae (purple sulfur bacteria) in the dry season in particular in Myrmidon Creek (IndVal 0.92, *P* < 0.001) while there were more cyanobacteria such as Phormidiaceae in the wet season (IndVal >0.84, *P* < 0.001). Year of sampling (2013 vs. 2014) was the second most important factor for East Arm and third for Shoal Bay after seasons and tides.

**Table 1:** PERMANOVA analysis of water microbiota.

	**East Arm**		**Shoal Bay**	
	**Pseudo F (df) ^#^**	**ECV**	**Pseudo F (df) ^#^**	**ECV**
Seasons (wet vs. dry)	47.4 (1) ^***^	0.19	23.5 (1) ^***^	0.17
Years (2013 vs. 2014)	23.3 (1) ^***^	0.13	12.2 (1) ^***^	0.12
Tides (spring vs. neap)	17.5 (1) ^***^	0.11	18.1 (1) ^***^	0.16
Creeks^*^	4.4 (2) ^**^	0.06	8.1 (2) ^***^	0.12
Sites nested in creeks	1.5 (8)	0.04	1.9 (9) ^*^	0.08
IA Years × Seasons	14.2 (1) ^***^	0.14	11.8 (1) ^***^	0.16
IA Years × Tides	10.0 (1) ^***^	0.12	5.6 (1) ^**^	0.11
IA Seasons × Tides	10.0 (1) ^***^	0.12	9.6 (1) ^***^	0.15
IA Years × Seasons × Tides	5.8 (1) ^**^	0.12	5.8 (1) ^**^	0.16
IA Creeks × Tides	6.7 (2) ^***^	0.11	4.8 (2) ^***^	0.12
Residual		0.18		0.24

PERMANOVA analysis of the water microbiota of East Arm and Shoal Bay using a cross design with fixed factors; creeks, years, seasons, tides and sites. Sites were nested in creeks. Interactions (IA) between factors are indicated for P < 0.01. “ECV” for square root estimates of the component of variation as an indication of the effect size independent of degrees of freedom and in the unit of the UniFrac distance matrix.

# Pseudo-F with degrees of freedom (df) and

*** *for strong evidence (P = 0.001)*,

** *good evidence (0.01 > P > 0.001)*,

* *evidence (0.05 > P > 0.01) – all tests with 995-999 permutations*.

* *Urban runoff and effluent-related samples were excluded from the PERMANOVA analysis*.

### The influence of tides and location

**Figure 2:**
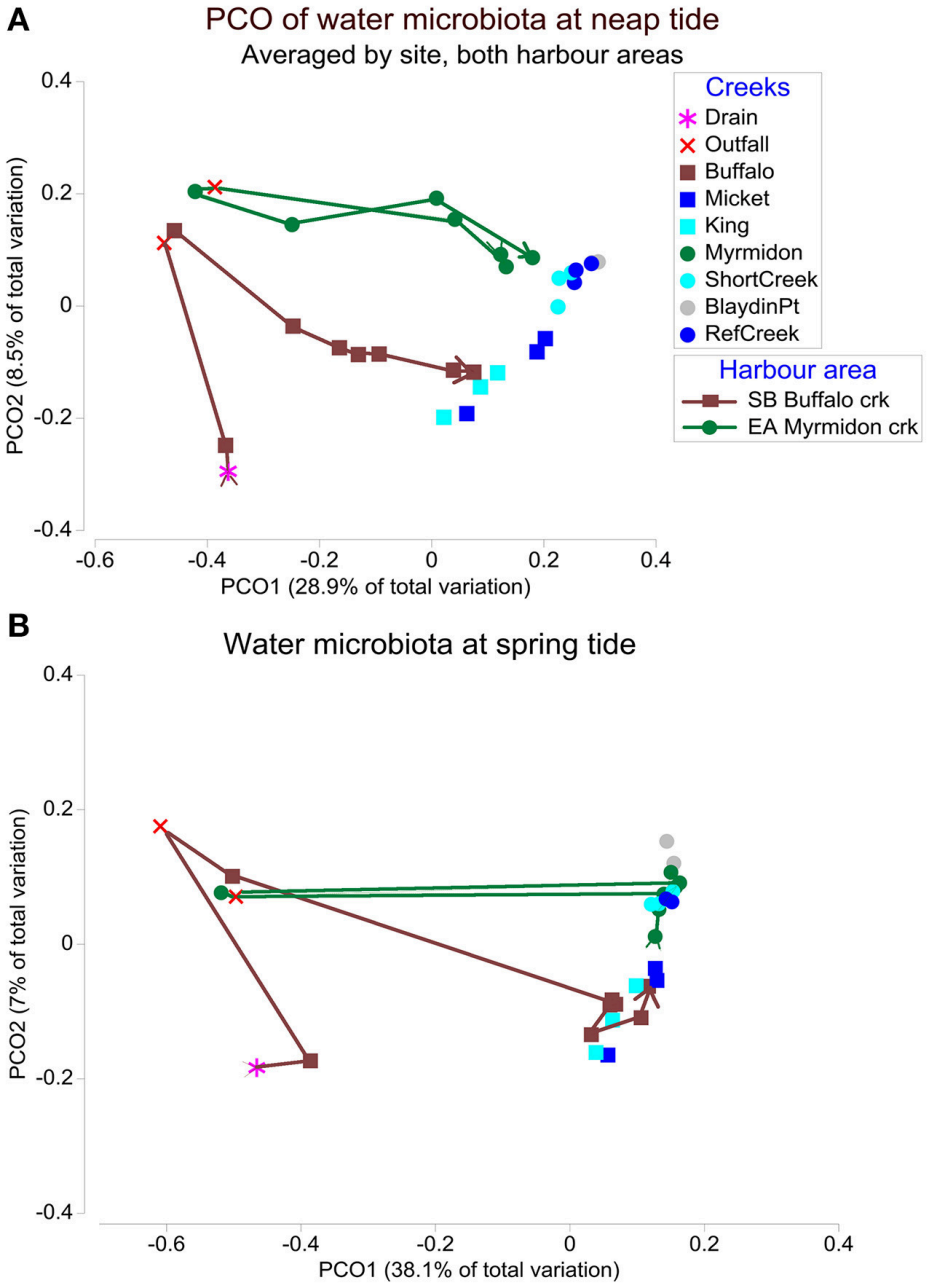
The water microbiota at neap and spring tides. Principal coordinate ordination (PCO) plots of the microbiota for East Arm and Shoal Bay water during **(A)** neap tides and **(B)** spring tides. The PCOs are based on a weighted UniFrac distance matrix of microbial OTUs averaged by site. The trajectories indicate Buffalo Creek (brown) and Myrmidon Creek (green) from sites upstream to the mouth. The first two PCO axes explained 37.4% of the microbial variation in **(A)** and 45.1% in **(B)**.

There was a strong effect of neap vs. spring tides and it was the second most important factor for Shoal Bay and third for East Arm (Table 1). There were significantly more Burkholderiales and Phormidiaceae during neap tides in particular for Shoal Bay and the wet season (IndVal >0.81, *P* < 0.001) while spring tides were associated with more Verrucomicrobia or Planctomycetes (IndVal >0.75, *P* < 0.001). There was a significant interaction between creeks and tides (Table 1) which indicated that tides also changed the effect of location upon the microbiota. There was a creek specific microbiota signature for neap tides, which was largely absent for spring tides (Figure 2). While the microbiota from the impacted creeks (Myrmidon and Buffalo) significantly differed from their reference creeks for neap and spring tides (*t*-test, *P* < 0.01), the reference creeks only differed from each other during neap tides. The creek-specific signature was particularly evident for Shoal Bay where the Buffalo Creek microbiota gradually changed from an impacted signature at the outfall to a Shoal Bay background signature that was also shared by the reference creeks King and Micket (Table 1, Figure 2). The bacteria in the impacted Myrmidon Creek showed a typical disturbance—recovery trajectory with the effluent entering the creek through a side-tributary and the microbiota reverting to the background signature close to where the tributary water entered the main channel (Figure 2). There was no significant difference in the microbiota dispersion between creeks (*P* > 0.1).

Comparing the microbiota between the two effluent outfalls, the effluent discharging into Buffalo Creek contained significantly more Deinococci, Planctomycetes and Chlamydiales (IndVal = 1 for all, *P* = 0.038), while the effluent at Myrmidon Creek had more Clostridiales, Cloacamonae and Bacteroidetes (IndVal > 0.9, *P* = 0.038). The urban runoff into the Buffalo Creek headwaters contained significantly more Enterococcaceae, Aeromonadaceae and Lactobacillales, compared to the effluent at Buffalo Creek (IndVal > 0.8, *P* < 0.01).

We tested the predictive ability of the microbiota for location i.e., given the microbial signature, was it possible to predict the creek from which the water was collected? In particular for Shoal Bay there was good separation between the impacted and most distant reference creek (Table S2). Buffalo Creek showed the highest correct classification rate with 80% while the water microbiota from Myrmidon Creek showed the lowest predictive ability with only 42% of samples correctly assigned. Control samples had a considerable amount of misclassifications between each other reflecting their similarity.

### The influence of abiotic factors on the water microbiota

#### The spatial and temporal distribution of abiotic factors

Water nutrients, salinity, pH, DO, turbidity and chlorophyll-a data are summarized in the Table S3. For Buffalo Creek, all nutrients showed a distinct gradient from high in the effluent discharge (median e.g., TDN 6,780 ppb, TDP 1,360 ppb) to lower levels at the creek mouth (median TDN 125 ppb, TDP 26 ppb). The urban runoff into Buffalo Creek headwaters had raised nitrate (median 250 ppb) and TDN levels (median 357 ppb) compared to the creek water. For Myrmidon Creek, no such distinct gradient was evident and only the site in the tributary close to the effluent discharge showed raised nutrient levels (Table S3). The effluent discharging into the Myrmidon Creek tributary showed particularly high TDN and ammonium levels (median 17,500 ppb and 5,650 ppb). While levels were considerably lower, all reference creeks had higher nutrient concentrations at the most upstream site compared to the mouth. This was most pronounced for Micket Creek (median TDN 217 ppb upstream vs. mouth 81 ppb) and least for Reference Creek in East Arm (TDN 92 ppb upstream vs. mouth 82 ppb).

A seasonal comparison of abiotic factors accounting for sites and years revealed an average 23% more TDN (*P* = 0.049), 63% more TSS (*P* = 0.003) and 76% higher turbidity (*P* < 0.001) in the wet season compared to the dry season. Similarly, a comparison of abiotic factors between neap and spring tides showed an average 2.5 times higher TDN levels (*P* < 0.001) and 1.9 times more TDP (*P* = 0.002) at neap tides while TSS levels were 1.7 times higher at spring tides (*P* = 0.005). There was a distinct separation of outfall related samples for both Shoal Bay and East Arm, marked by high levels of nutrients, chlorophyll-a, turbidity and lower levels of dissolved oxygen and salinity (Figure S4).

#### The water microbiota and temperature, dissolved oxygen and season

The East Arm water microbiota showed a season-specific clustering, and in particular, a distinct cluster for the last dry season in 2014 (Figures S4, S5), which was mainly associated with lower water temperature. The water was colder in the last round with average 25.5°C compared to 30.1–30.4°C for the other three sampling rounds (Dunn's test *P* < 0.001). Despite Shoal Bay recording the same water temperature no such round- or season-specific clustering was evident (Table 2, Figure S5). Dissolved oxygen (DO) was significantly higher in the last sampling round with average 89.4% compared to 74.4–83% for the other rounds (Dunn's test *P* < 0.001, Figure S5). Similarly to temperature, DO played a more important role for the East Arm microbiota compared to Shoal Bay where its overall impact was negligible (Table 2).

**Table 2:** Abiotic factors and the water microbiota.

	**Marginal test**		**Sequential test accounting for other factors in the multivariate linear model**		
	Pseudo-F ^***^	**% proportion explained^#^**	**Pseudo-F**	**% proportion explained ^#^**	**% cumulative prop explained**
**(A) EAST ARM**					
N-NH_4_^+^	55.4	28.8	55.4 ^***^	28.8	28.8
Temp	13.4	8.9	19.4 ^***^	8.9	37.7
TDP	51.6	27.3	15.6 ^***^	6.4	44.1
Salinity	40.8	22.9	10.0 ^***^	3.8	48.0
DO	15.2	10.0	7.9 ^***^	2.9	51.0
DOC	54.5	28.4	4.6 ^***^	1.6	52.6
Turb	19.0	12.2	4.3 ^***^	1.5	54.2
Chl-a	55.1	28.7	3.6 ^***^	1.2	55.4
P-PO_4_^3−^	40.1	22.7	2.2 ^*^	0.7	56.2
pH	5.6	3.9	1.9 ^*^	0.6	56.9
DistOF^∧^	21.0	13.3	1.9 ^*^	0.6	57.5
TDN	59.7	30.3	These factors did not improve the multivariate model fit		
Depth	35.2	20.4			
N-NO_2_	14.1	9.3			
N-NO_3_	6.7	4.6			
**(B) SHOAL BAY**					
Salinity	57.6 ^***^	30.2	57.6 ^***^	30.2	30.2
N-NO_2_	24.2 ^***^	15.4	19.3 ^***^	8.9	39.1
N-NO_3_	39.4 ^***^	22.8	6.6 ^***^	2.9	42.0
DOC	26.7 ^***^	16.7	6.3 ^***^	2.6	44.7
Depth	36.5 ^***^	21.5	6.1 ^***^	2.5	47.2
Temp	8.0 ^***^	5.7	5.5 ^***^	2.1	49.4
Turb	5.9 ^***^	4.2	4.1 ^***^	1.6	51.0
N-NH_4_^+^	31.7 ^***^	19.2	3.9 ^***^	1.4	52.5
pH	4.3 ^**^	3.1	2.8 ^***^	1.0	53.6
DistOF^∧^	15.1 ^***^	10.2	2.7 ^**^	1.0	54.6
DO	4.8 ^**^	3.5	2.7 ^***^	0.9	55.6
TDN	46.0 ^***^	25.7	2.7 ^***^	0.9	56.6
TDP	29.1 ^***^	18.0	2.8 ^***^	0.9	57.6
Chl-a	24.8 ^***^	15.7	This factor did not improve the model fit		

*Distance-based linear model for **(A)** East Arm and **(B)** Shoal Bay showing the Pseudo-F and proportion explained for the abiotic factors in marginal and multivariate linear tests with the latter accounting for the other factors in the model. The order of factors is according to their importance in the multivariate model*.

*** *P value = 0.001 [applies to all marginal tests for **(A)** East Arm]*,

** *0.01 < P value < 0.001*,

* 0.05 < P value < 0.01;

# *Proportion of water microbiota data explained by abiotic factor*;

∧ *DistOF distance to outfall*.

#### The water microbiota and salinity

Salinity was the most important abiotic factor for the Shoal Bay water microbiota (Table 2). This was in contrast to East Arm where temperature and various nutrients explained more of the microbiota variation (Table 2). Salinity levels were significantly higher in the dry season for both harbor areas with a median 37.0 compared to wet season median 31.0 (East Arm) and 20.7 for Shoal Bay (Mann-Whitney test, *P* < 0.001). Shoal Bay not only showed a larger salinity difference between seasons but also along the creeks from the upper regions to the mouth, and this was evidenced by a long salinity vector in the Shoal Bay dbRDA. The salinity gradient was most pronounced during the wet season with a median increase from upstream 10.6 to 30.3 at the mouth of the creeks in Shoal Bay. No such strong salinity gradient was found in East Arm (wet season upstream 26.3 to mouth 28.5). The strong salinity gradients for Shoal Bay creeks in the wet season also contributed to the large differences in the composition of the microbiota within a creek. During that time the neap water microbiota within a Shoal Bay creek was more heterogeneous (weighted UniFrac distance 0.54–0.58) compared to the dry season (within-creek distance 0.32–0.39). The East Arm water microbiota was generally more homogenous across both seasons (within-creek distance 0.33–0.41).

#### The water microbiota and nutrients

Nutrients played a significant role in explaining the microbiota variance. For East Arm, ammonia explained most of the microbiota variance with 28.8% in the marginal and multivariate model followed by temperature and TDP (Table 2). The importance of ammonia in the model reflected the strong ammonia gradient with a median 16,300 ppb at the Myrmidon outfall where the effluent discharged into the mangroves compared to median 11 ppb in the reference creeks. Ammonia played a lesser role for the Shoal Bay microbiota model and after salinity, nitrite and nitrate accounted for the major part of the explainable variance of the Shoal Bay microbiota (Table 2). NO_x_ levels were considerably higher in the effluent discharged into Buffalo Creek compared to the effluent at Myrmidon Creek but levels were also raised in the Buffalo Creek urban runoff (Table S3).

**Figure 3:**
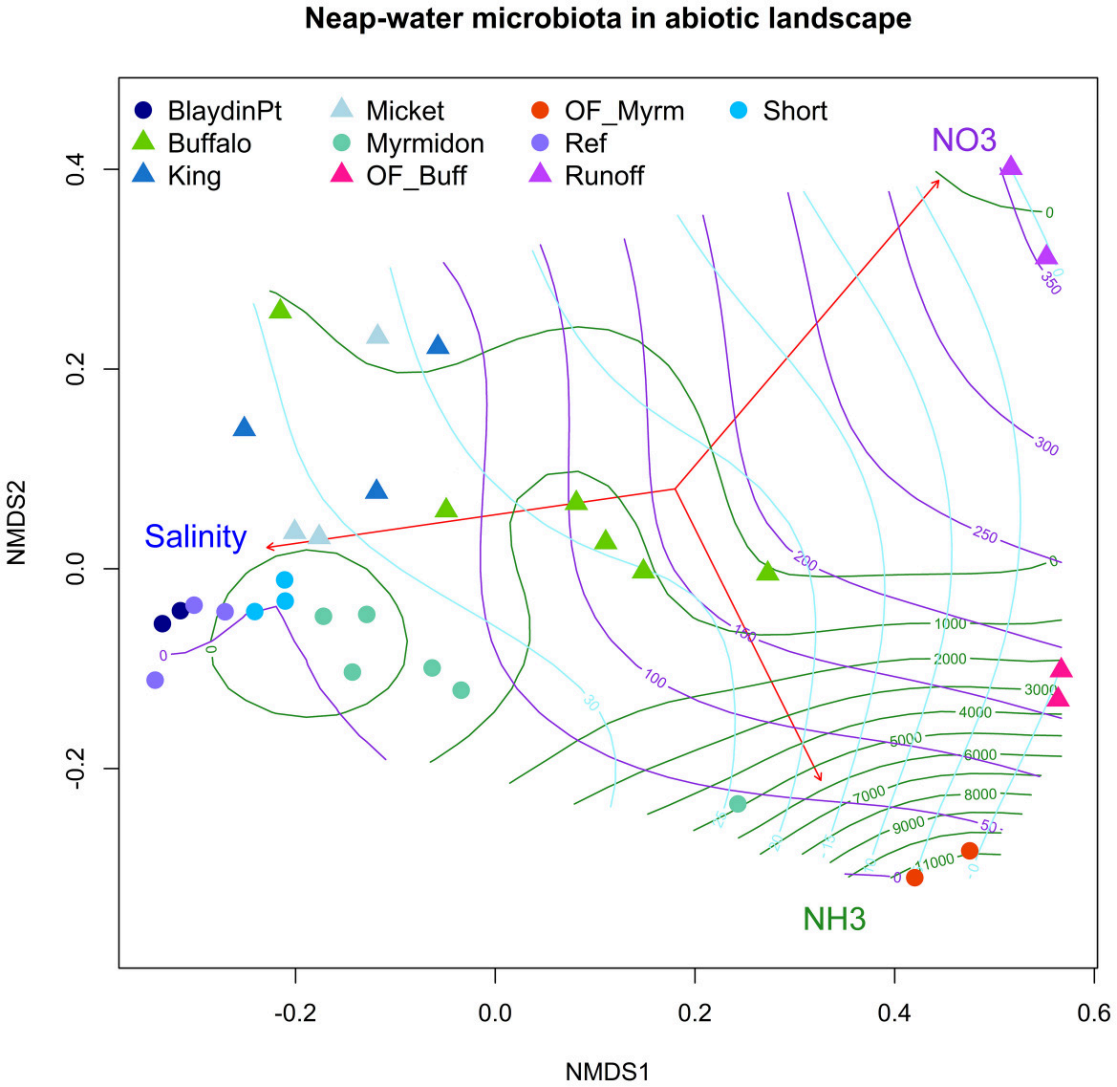
Water microbiota in the nutrient landscape. A nMDS ordination on the site-averaged weighted UniFrac distance matrix of the neap water microbiota in Shoal Bay and East Arm. The function envfit (library vegan in R) was used to plot the correlation vectors between nitrate, ammonia, salinity and the nMDS axes. The contour-lines mark the ammonia (green), nitrate (violet) and salinity (light blue) landscape which was calculated using a non-linear generalized additive model and thinplate spline interpolation implemented in the function ordisurf of the library vegan in R.

The contrast in the gradients of salinity, nitrate and ammonia in East Arm and Shoal Bay and their association with the microbiota is illustrated in Figure 3. It shows the fitted topography of these three abiotic factors across the site-averaged microbiota landscape with highest nitrate levels for the urban runoff at Buffalo Creek and highest ammonia levels for the site in the mangroves at the Myrmidon effluent outfall. The nutrient topography for Buffalo Creek reflected the slower reversion of the microbiota to background levels. This was in contrast to Myrmidon Creek for which only the tributary close to the outfall had markedly raised nutrient levels and a distinct microbial profile. Each of TDP, DOC, and TDN explained more than 27–30% of the microbiota in East Arm and 17–26% in Shoal Bay in the marginal tests. However, in the multivariate model they accounted for considerably less variance in the microbiota data due to their collinearity with NO_x_ and/or ammonia (Table 2, Figure S5).

### Variation partitioning of the water microbiota into spatial and abiotic contributions

**Figure 4:**
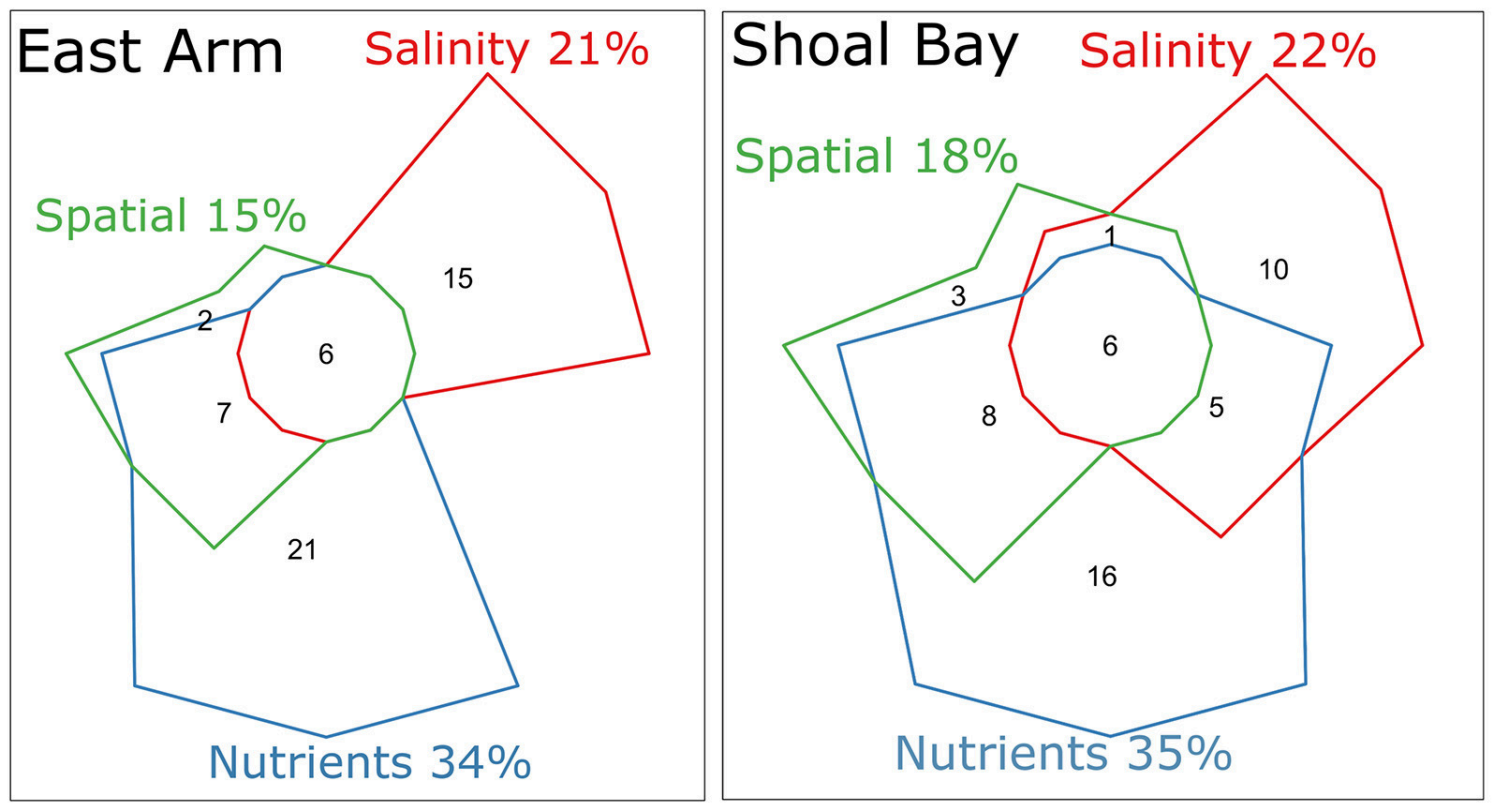
Variation partitioning of the water microbiota at neap tide from East Arm and Shoal Bay. The numbers indicate percentage of the microbiota variation explained by the corresponding fraction or combination thereof. Percentages are based on the adjusted R^2^ accounting for the number of other predictors. Nutrients and salinity were standardized to the same scale. The forward selected nutrients explaining significant parts of the microbiota in a redundancy analysis included ${\text{PO}}_{4}^{3-}$, ${\text{NH}}_{4}^{+}$, NO_3_, NO_2_, DOC, and TOC for East Arm and TP, TDP, ${\text{PO}}_{4}^{3-}$, TDN, ${\text{NH}}_{4}^{+}$, NO_3_, NO_2_, DOC for Shoal Bay. The spatial fraction consisted of the PCNM eigenvectors. The fraction shared between salinity and nutrients was minus 3.6% for East Arm and assumed zero. The residual unexplained fraction for East Arm was 52.6 and 50.8% for Shoal Bay.

An eigenvector-based spatial analysis of the neap water microbiota of Shoal Bay showed three independent patterns of which two consisted of linear trends along the x and y axes reflecting the effluent point source at Buffalo Creek (Figure S6). The third non-linear pattern only explained 2.2% of the Shoal Bay microbiota variance and showed a correlation between the urban runoff microbiota into the upper reaches of Buffalo Creek and the most upstream Micket site. Micket Creek also receives urban runoff albeit to a much lesser degree. The last pattern was associated with higher DOC, TN, and TP for these upstream sites (Mann-Whitney *U*-test, *P* < 0.01). There were significantly more Neisseriaceae in these upstream samples (IndVal 0.8, *P* value 0.01). For East Arm, there was only one spatial pattern significantly associated with the neap water microbiota. It explained 14.9% of the microbiota variance and reflected the effluent outfall at Myrmidon (Figure S6). Both harbor areas showed similar variation partitioning patterns. Nutrients accounted for most of the explainable variation in the microbiota with total 34–35%, followed by salinity with 21–22%, and spatial components 15–18% (Figure 4). While 5% of the Shoal Bay microbiota variance was explained by concomitant local changes in salinity and nutrients, there was no such measurable fraction for East Arm. The overall contributions of the spatial fraction were smaller for the East Arm model and only a quarter of the salinity and nutrient fractions were also shared with the spatial fraction compared to a third for Shoal Bay.

### Relatedness of water microbiota across darwin harbour

**Figure 5:**
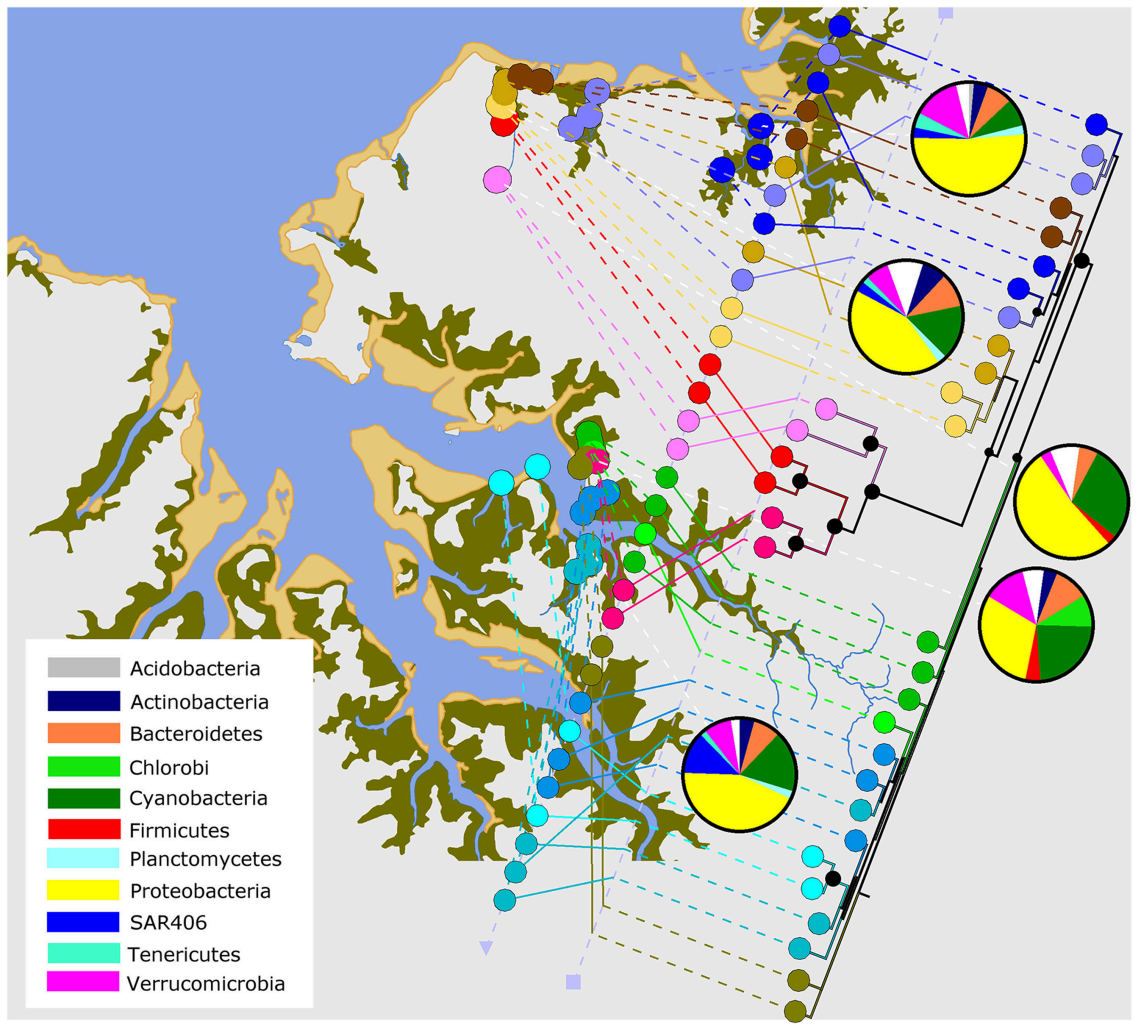
Microbiota relatedness across the harbor. Relatedness of the microbiota in Shoal Bay and East Arm based on a neighbor-joining tree on the weighted UniFrac distance matrix of median water OTU counts per site. A Bootstrap analysis was conducted on 500 resampled trees. Nodes with a black circle indicate >99% support and smaller circles >75% support. The tree and pie charts of main bacterial phyla (white for other) were combined with a Darwin Harbour map using GenGIS 2.4.0 (Parks et al., 2013).

In support of the findings above, the Shoal Bay microbiota had a stronger site-specific structure compared to East Arm (Figure 5). Shoal Bay and in particular the Buffalo Creek microbiota were more similar to the treated effluent and urban runoff related microbiota than the East Arm microbiota. A comparison of bacterial phyla between sites showed the distinct higher abundance of Firmicutes in the effluent and runoff microbiota, and green sulfur bacteria of the phylum Chlorobi in the effluent (Figure 5). Marine group A (SAR406) was distinctively more abundant in East Arm water compared to Shoal Bay water.

### The sediment microbiota

#### The sediment vs. the water microbiota

The microbiota of the sediment significantly differed from the water microbiota (Pseudo-F 171, *P* = 0.001) with an average dissimilarity of 0.75 between these groups. There were more bacteria of the phyla Acidobacteria, Caldithrix, or Nitrospirae in sediment while water contained more Actinobacteria, Bacteroidetes, or Verrucomicrobia (Kruskal Wallis test, *P* < 0.001) (Figure S7). There was no difference in the Simpson diversity between impacted and control samples for sediment (Figure S8). This was in contrast to the water alpha diversity. For East Arm water samples, effluent from the outfall had significantly lower diversity if compared to impacted or control samples [Kruskal-Wallis ${\text{chi}}_{\left(2\right)}^{2}$ = 10.8, *P* = 0.005] (Figure S8). For Shoal Bay water, the urban runoff had a significantly higher evenness and Simpson diversity if compared to impacted or reference creek samples [Kruskal-Wallis ${\text{chi}}_{\left(3\right)}^{2}$ = 10.2, *P* = 0.017] (Figure S8).

Overall, the sediment microbiota was more homogenous than the water microbiota and temporal and spatial patterns were less distinct. The sediment microbiota differed between years for both harbor areas similar to the water microbiota, but in contrast to water there was only a weak seasonal effect (Table S4).

In contrast to the neap water microbiota, the site-averaged sediment bacterial community showed no clear creek and location-specific patterns except if close to the outfalls (Figure S9). There was no difference in the sediment microbiota between East Arm creeks (Table S4, Figures S9, S10). In contrast, there was a creek-specific signal for the Shoal Bay sediment (Table S4, Figures S9, S10) and there were high correct classification rates between 89 and 94% for King, Micket and Buffalo Creek sediment (Table S4). Bacteria of the family Desulfobacteraceae, order Nitrosomonadales or Clostridiales were more abundant in Buffalo Creek sediment compared to Micket and King Creek sediment while the latter two creeks contained more Rhodospirillaceae or Gemmatimonadetes and King Creek sediment had the highest counts of Nitrospirales (Kruskal-Wallis test, *P* < 0.001).

#### The sediment microbiota and abiotic factors

Most sediment samples consisted of mud i.e., clay and silt with small grain size. Aluminum levels were used as a proxy for grain size (Din, 1992). Sediment from East Arm generally had higher levels of Al than Shoal Bay sediment indicating smaller grain size i.e., more clay. Sediment ORP levels significantly differed between creeks (Kruskal-Wallis test, *P* = 0.008). King and Reference Creek showed the highest ORP levels (mean −60 mV) and Buffalo and Myrmidon the lowest (−156 and −173 mV). Cu and Zn proved important abiotic factors explaining the sediment microbiota for both harbor areas (Figures S11, S12). For East Arm, Cd, Zn, Cu and *P* each explained more than 30% of the sediment microbiota independently in marginal tests (Figure S11). ORP tended to be less negative or positive in the reference creek sediment indicating a more oxidizing environment compared to the impacted creeks. For Shoal Bay, the top abiotic factors Zn, Pb, ORP, TKN, Cu, TOC, Ca, and S each explained 10–11% of the sediment microbiota in marginal tests (Figure S12). TKN and sulfur levels were raised in the sediment close to the outfall and in upper Buffalo Creek. However, similar to the marginal tests, the multivariate model did not explain the data well and the first two dbRDA or CCA axes only explained 27 or 16% of the total microbiota variation (Figures S11, S12).

## Discussion

We analyzed the temporal and spatial patterns of water and sediment microbiota of a macrotidal mangrove-lined estuary in the wet-dry tropics. The water microbiota provided clearer spatial and temporal patterns compared to the sediment microbiota which was more uniform. Since the primary aim of this work was to identify a spatial human-impact signal, we focused on the water microbiota for those analyses involving measures of human-impact.

### The water microbiota

Temporal factors showed the strongest relationship with the water microbiota. Temporal shifts in the microbiota composition of temperate aquatic habitats have been linked to seasonal variation of physicochemical water properties (Fuhrman et al., 2015) and allochthonous inputs upon increased rainfall (Jeffries et al., 2016). In this study, the dry and wet seasons showed the strongest association with the water bacteria composition. The monsoonal wet season brings high rainfall, which significantly decreases salinity levels while raising TDN and TSS levels. More Phormidiaceae were detected in the wet season, a family of blue-green algae which also contains the genus *Planktothrix* with members able to form toxic blooms in freshwater habitats (Hossain et al., 2012). In Malaysia and Bangladesh, differences in phytoplankton diversity between monsoonal seasons were found to be dependent on runoff, nutrients, TSS and temperature (Sidik et al., 2008; Hossain et al., 2012). In the dry season, significantly more phototrophic purple sulfur bacteria of the family Chromatiaceae were detected. Members of this family have been previously described from this region (Cornall et al., 2013), and they primarily occur in stagnant water (Pfennig and Trüper, 1992), conditions more likely in the dry season when there is virtually no freshwater input.

The dry season was also associated with lower water temperature and more dissolved oxygen. Intriguingly, while both areas, East Arm and Shoal Bay recorded the same water temperature, only the water microbiota in East Arm was associated with changes in water temperature and dissolved oxygen. Water in East Arm can be trapped for several months with high evaporation in the dry season leading to inverse estuary effects and increased salinity (Williams et al., 2006; Tonyes et al., 2015). While the residence time for the whole of East Arm is long, the creeks feeding East Arm are open channels allowing good mixing of water within East Arm, which weakens spatial patterns and dilutes nutrients from effluent and stormwater. Thus, considering long water residence times and no distinct nutrient and salinity gradients, temporal seasonal patterns might become more pronounced and may explain the clear seasonal clustering of the East Arm microbiota.

In contrast to East Arm, spatial patterns including the association with salinity were decisive for the Shoal Bay water microbiota. Salinity gradients from the upper reaches to the mouth of the creeks were considerably larger compared to East Arm, particularly in the wet season with more freshwater inputs into the headwaters of Shoal Bay. The main reason for the more pronounced salinity gradient across Shoal Bay was likely reduced mixing within the creeks as sand bars at the mouths of the creeks restrict tidal water exchange and wave mixing. These sand bars act as weirs forming barrier bar estuaries—a phenomenon not seen in East Arm. A particularly large intertidal sand bar at the mouth of Buffalo Creek reduces the tidal amplitude by up to 50% (Smith et al., 2012). Flushing of Buffalo Creek is further impeded by the creek's narrow meandering morphology which creates eddy circulations that restrict longitudinal mixing. In the dry season, the upper reaches of Micket and King Creeks turn hypersaline with levels up to 41 ppt (Ridd and Stieglitz, 2002). The Shoal Bay catchment also has extensive areas of salt pans which are regularly inundated at high tides. In relation to these salinity gradients, the Shoal Bay microbiota were more heterogeneous within a creek compared to East Arm, and had more location specific signatures, particularly in the wet season.

Location or creek specific microbial patterns were mainly present at neap tides and virtually absent during spring tides when the water was well mixed. Exceptions were sites close to the effluent outfall and urban runoff. Differences between neap and spring tides were the second most important factor for the Shoal Bay water microbiota after seasons, and ranked third for East Arm after seasons and sampling years. Macro-tides and wet season run-off both potentially represent major challenges to microbiota survival, and we know relatively little about how they respond to shifts in salinity. In a similar setting (Hyun et al., 1999) reported that the microbial community alternated between autochthonous halotolerant estuarine and allochthonous halophobic freshwater populations. We found that Verrucomicrobia, abundant in marine environments (Freitas et al., 2012), were more abundant during spring tides, which suggests increased exchange with oceanic water. In contrast, neap tide conditions during the wet season in Shoal Bay were associated with more Burkholderiales and Phormidiaceae, which reflects the higher freshwater inputs and reduced mixing with marine water. The higher abundance of blue-green algae of the Phormidiaceae family might also reflect the elevated nitrate levels in Shoal Bay (Heath et al., 2016). The finding of clearer spatial patterns of the water microbiota during neap tides suggests that water quality monitoring at neap tides better reflects the local water conditions including anthropogenic nutrient sources. We also found higher nutrient levels at neap tide compared to spring tides. It was of note that the composite of nutrient species showed fewer spatial patterns than the neap water microbiota suggesting the latter to be a superior tool to discern the spatial impact of elevated nutrients.

Nutrient levels were important for explaining the water microbiota for both harbor areas. For the East Arm water microbiota, TDN accounted for most of the microbiota variance in marginal tests, and ammonia was the most important factor in the multivariate distance model. This reflected the high ammonia levels measured in the effluent at the Myrmidon outfall, which were diluted rapidly once reaching the Myrmidon main channel. The Simpson diversity of the effluent microbiota at the Myrmidon outfall was significantly lower than at any other site in this study including the effluent microbiota at the Buffalo Creek outfall. A decrease in microbial diversity has been associated with a decline in ecosystem function (Sun et al., 2012). The water microbiota showed a disturbance-recovery trajectory along Myrmidon Creek with a rapid reversal to the background microbiota after the effluent entered the main channel through the tributary, and the rest of the East Arm water microbiota clustered tightly. This pattern also reflects the increased water mixing and low nutrient levels within East Arm compared to Shoal Bay.

In contrast to East Arm, TDN and NO_x_ explained most of the microbiota variation in Shoal Bay after salinity. NO_x_ levels were considerably higher in the effluent discharged into Buffalo Creek compared to the Myrmidon outfall although these levels were still lower than those of ammonia. Deinococci and Planctomycetes bacteria were more abundant at the Buffalo outfall compared to the Myrmidon outfall. Both these taxa contain ammonia-oxidizing bacteria and have also been described in a batch reactor with high NO_2_ concentrations (Tan et al., 2008).

Indicative of the reduced water flushing in Buffalo Creek, there was only a gradual change of the water microbiota from the disturbed signature at the outfall back to the Shoal Bay background microbial composition at the mouth of the creek. Similarly, phylogenetic analysis of the water microbiota showed that the urban runoff and outfall microbial signatures from both outfalls were more similar to the microbial signatures from Shoal Bay water than to East Arm water. The urban runoff showed the highest nitrate concentration of all water samples. Nitrate is often the most common soluble nitrogen species in stormwater with various sources including soil nitrification processes, fertilizers, animal waste or abandoned landfills (Wakida and Lerner, 2005). The urban runoff also had the highest bacterial Simpson diversity and compared to the effluent at Buffalo outfall, contained more bacteria of families with known fecal indicators, commensals or human pathogen indicators such as Enterococcaceae, Lactobacillales, or Aeromonadaceae (Cabral, 2010; Roslev and Bukh, 2011).

The water microbiota differed at sites closer to the outfall and urban runoff regardless of seasons and tides. However, it was not clear how much this was due to eutrophication i.e., nutrient load and/or changes in salinity levels due to freshwater inputs. We therefore conducted a variation partitioning analysis on the neap water microbiota for each harbor area to distinguish between nutrient, salinity, and spatial factors. A spatial analysis showed that the microbiota changed with increasing distance from the effluent point source. A non-linear spatial trend also showed an association between the microbiota at the most upstream Buffalo and Micket Creek sites. Both these creeks receive urban runoff and both had significantly more Neisseriaceae than at other sites. Neisseriaceae have been described in groundwater of agricultural land and contain several mammalian commensals and pathogens (Wakelin et al., 2011). For both harbor areas, the composite of nutrient species accounted for the major part of the explainable variation in the water microbiota. The variation partitioning for East Arm showed a distinct lack of a fraction which associated the microbiota variance with salinity and nutrient levels. This indicates that local changes in salinity and nutrients were not well correlated and may reflect the generally low nutrient levels across East Arm with the largest changes in nutrients confined to the Myrmidon outfall and adjacent side-tributary. Compared to Shoal Bay, the spatially structured environmental variation (Borcard et al., 2011) as well as the total spatial fraction explained less of the East Arm microbiota variance. This again reflects the less site-specific bacterial signatures for East Arm.

### The sediment microbiota

Mangrove ecosystems are highly productive with a diverse sediment microbial community recycling nutrients and acting as a carbon and nitrogen sink (Holguin et al., 2001). The sediment microbiota in this study also showed a considerably larger diversity than the surface water microbiota. The sediment and water microbiota composition significantly differed which was also due to sediment pore and benthic water typically being anoxic with associated significant changes to redox sensitive reactions. There was a distinct absence of spatial patterns for the sediment microbiota in East Arm, which did not differ beyond the immediate outfall confirming the still healthy condition of the sediment in this part of the harbor. In contrast, the sediment microbiota in Shoal Bay showed a creek-specific signature in particular for Buffalo Creek. The creek has received effluent and urban runoff for over 40 years and sediment pore water is enriched with dissolved inorganic nutrients. Benthic nutrient fluxes are high while denitrification efficiencies are variable including below 10% (Smith and Haese, 2009; Smith et al., 2012). Increased tidal pumping and exchange with nutrient enriched sediment pore water during spring tides can further increase nutrient loads in surface water peaking at the following neap tide (Call et al., 2015). This exchange is further enhanced through numerous crab burrows in the mangrove sediment increasing hydraulic connectivity and the surface area of the sediment water interface. Also pelagic primary production rates were found to be of 1–2 order of magnitude higher in Buffalo Creek than in the reference creeks (Smith et al., 2012). The discharge of effluent, exacerbated by limited creek flushing, leads to increased algal biomass and degraded water quality with hypoxic conditions in particular at night (Smith et al., 2012). We found more anaerobic sulfate reducing Desulfobacteraceae and nitrifying Nitrosomonadales in the sediment of Buffalo Creek, as well as Chloroflexi bacteria which contain green non-sulfur bacteria and Clostridiales, an order of mostly fecal anaerobes with known sewage indicators (McLellan et al., 2013). These bacteria have previously been described in polluted sediment receiving sewage effluent (Zhang et al., 2008; Lu and Lu, 2014).

For both treated sewage outfalls, sediment microbiota were associated with higher levels of copper in sediment which is consistent with other reports for sewage discharge sites in Darwin Harbour (Padovan et al., 2012). The bacteria in upper Buffalo Creek sediment were associated with higher levels of sulfur, which could explain the greater detection of Desulfobacteraceae in this creek. In King Creek, sediment bacteria were linked to a more positive redox potential reflecting the more oxygenated and less nutrient rich environment. It also contained more of the nitrite oxidizing Nitrospirales which have been found to decrease in abundance under anoxic conditions in marine sediment (Devereux et al., 2015). Despite the creek-specific signature of the Shoal Bay sediment microbiota and association with some abiotic factors, they explained a considerably smaller amount of the sediment microbiota compared to the water microbiota for both harbor areas. This is indicative of sediment acting as a long-term sink for nutrients and reflecting historical as well as current water conditions. While for many ecosystems, contemporary environmental conditions are crucial for bacterial species sorting, other studies including on sediment found a slow response of the microbiota to changing conditions and past processes were equally important to shape a bacterial community (Reed and Martiny, 2013; Andersson et al., 2014).

## Conclusions

Temporal drivers, namely seasons and tides, had the strongest relationship to the water microbiota, which reflects the macrotidal nature of the estuary and its location in the seasonally extreme wet-dry tropics. While the sediment microbiota reflected current and past water conditions, the neap-tide water microbiota provided the clearest spatial discrimination of the current water conditions, and thus, might be best suited for water quality monitoring. There were variations in patterns and drivers of the microbiota between the two harbor areas reflecting its complex hydrodynamics. Despite this, the microbial community consistently differed at sites close to point source inputs of treated sewage effluent and urban stormwater, and the composite of nutrient levels explained more of the microbial variation than did salinity. This work has laid the groundwork for further studies to identify bio-indicators suitable for routine monitoring to measure human impacts in complex ecosystems.

## Author contributions

Study design: KG, NM, MK, KM, and MM. Study conduct: MK, AS, MM, KK, and NM. Data analysis: MK and KM. Data interpretation: MK, DW, KG, and NM. Paper writing: MK, KG, NM, KK, KM, AS, and DW.

### Conflict of interest statement

The authors declare that the research was conducted in the absence of any commercial or financial relationships that could be construed as a potential conflict of interest.
