# Supplementary material for: Spatial and Temporal Microbial Patterns in a Tropical Macrotidal Estuary Subject to Urbanization
Source: Front Microbiol. 2017 Jul 13;8:1313. doi: 10.3389/fmicb.2017.01313 (PMC5507994; doi:10.3389/fmicb.2017.01313)
Supplement: Supplementary file 1 [file Image1.PDF]

S1 Choice of sequencing pipeline and subsampling

The MOTHUR (Schloss et al., 2009) (v 1.34.1), UPARSE (Edgar, 2013) (64-bit v8), QIIME (Caporaso et al., 2010) (v 1.9.0) closed and open reference MiSeq SOP pipelines were run in a linux environment on the high performance computer at Charles Darwin University using the default parameters unless otherwise stated.

A uchime chimera-removal step was included in all pipelines (Edgar et al., 2011), the minimum identity for an OTU cluster was 97% for all pipelines and singleton OTUs were discarded. The Greengenes database was used for taxonomy assignment (release May 2013)(McDonald et al., 2012).

The suitability of these pipelines for our data (277 estuarine water and 192 sediment samples) was compared using the criteria in Table S1 and Figure S1. Subsampling to 17,000 sequences vs no subsampling were also compared as well as different distance matrices i.e. the Bray Curtis (on raw OTU data) vs. weighted Unifrac distance matrix (Table S1)(Figure S1).

Macrotidal tropical mangrove estuaries are a poorly characterised environment and accordingly, 44% of reads were discarded with the QIIME closed reference pipeline due to no taxonomic assignment using the Greengenes database (Table S1). Therefore, this pipeline was not followed further.

While the total number of OTUs was 8.2 times higher for the QIIME open reference pipeline compared to the UPARSE pipeline, their weighted Unifrac distance matrices were still highly correlated based on rank dissimilarities with a Spearman correlation of 0.94 (Figure S1). Both pipelines showed a similar explained variance in an unconstrained PCO (Table S1) with the weighted Unifrac and subsampling to 17,000 sequences showing the highest explained variance (Table S1).

The QIIME open reference was chosen over the UPARSE pipeline as its weighted Unifrac distance matrix was a good representative of the other tested approaches (Figure S1) and the PCO of its weighted Unifrac matrix showed a slightly higher explained variance (Table S1).

Subsampling to 17,000 sequences was performed with reasons outlined in the results of the main text.

The weighted Unifrac distance measure was chosen as it was less susceptible to change upon subsampling showing a correlation above 0.99 between the distance matrices based on all or subsampled sequences (Figure S1) . Furthermore, the PCO of the weighted Unifrac matrices also showed a higher explained variance than those generated from a Bray Curtis dissimilarity matrix (Table S1).

Figure S1: Second-stage dissimilarity matrix comparison

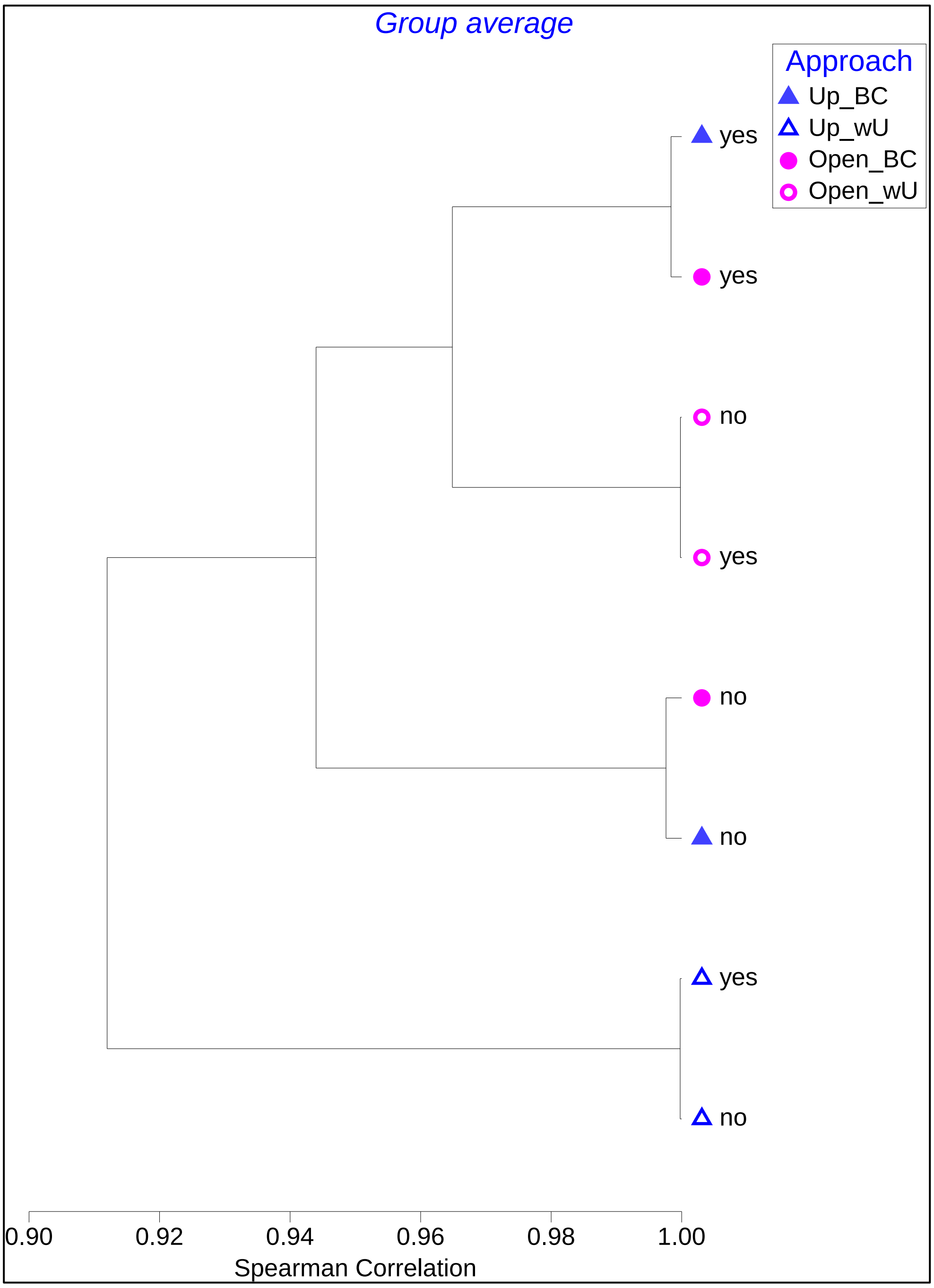

Figure S1 Legend: Hierarchical cluster analysis using group average of nonparametric Spearman correlations between distance matrices comparing different pipelines, subsampling and type of distance matrix. "Up" UPARSE pipeline, "Open" QIIME open reference pipeline, "BC" Bray Curtis distance matrix (on raw OTU data), "wU" weighted Unifrac distance matrix, "yes" subsampled to 17,000 sequences per sample, "no" no subsampling

Reference List

Caporaso, J.G., Kuczynski, J., Stombaugh, J., Bittinger, K., Bushman, F.D., Costello, E.K., et al. (2010). QIIME allows analysis of high-throughput community sequencing data. *Nat Methods* 7(5)**,** 335-336. doi: 10.1038/nmeth.f.303.

Edgar, R.C. (2013). UPARSE: highly accurate OTU sequences from microbial amplicon reads. *Nature Methods* 10(10)**,** 996-+. doi: 10.1038/Nmeth.2604.

Edgar, R.C., Haas, B.J., Clemente, J.C., Quince, C., and Knight, R. (2011). UCHIME improves sensitivity and speed of chimera detection. *Bioinformatics* 27(16)**,** 2194-2200. doi: 10.1093/bioinformatics/btr381.

McDonald, D., Price, M.N., Goodrich, J., Nawrocki, E.P., DeSantis, T.Z., Probst, A., et al. (2012). An improved Greengenes taxonomy with explicit ranks for ecological and evolutionary analyses of bacteria and archaea. *Isme Journal* 6(3)**,** 610-618. doi: 10.1038/ismej.2011.139.

Schloss, P.D., Westcott, S.L., Ryabin, T., Hall, J.R., Hartmann, M., Hollister, E.B., et al. (2009). Introducing mothur: Open-Source, Platform-Independent, Community-Supported Software for Describing and Comparing Microbial Communities. *Applied and Environmental Microbiology* 75(23)**,** 7537-7541. doi: 10.1128/Aem.01541-09.
