# Supplementary material for: Spatial and Temporal Microbial Patterns in a Tropical Macrotidal Estuary Subject to Urbanization
Source: Front Microbiol. 2017 Jul 13;8:1313. doi: 10.3389/fmicb.2017.01313 (PMC5507994; doi:10.3389/fmicb.2017.01313)
Supplement: Supplementary file 2 [file Image2.PDF]

Figure S2 Rarefaction Curves

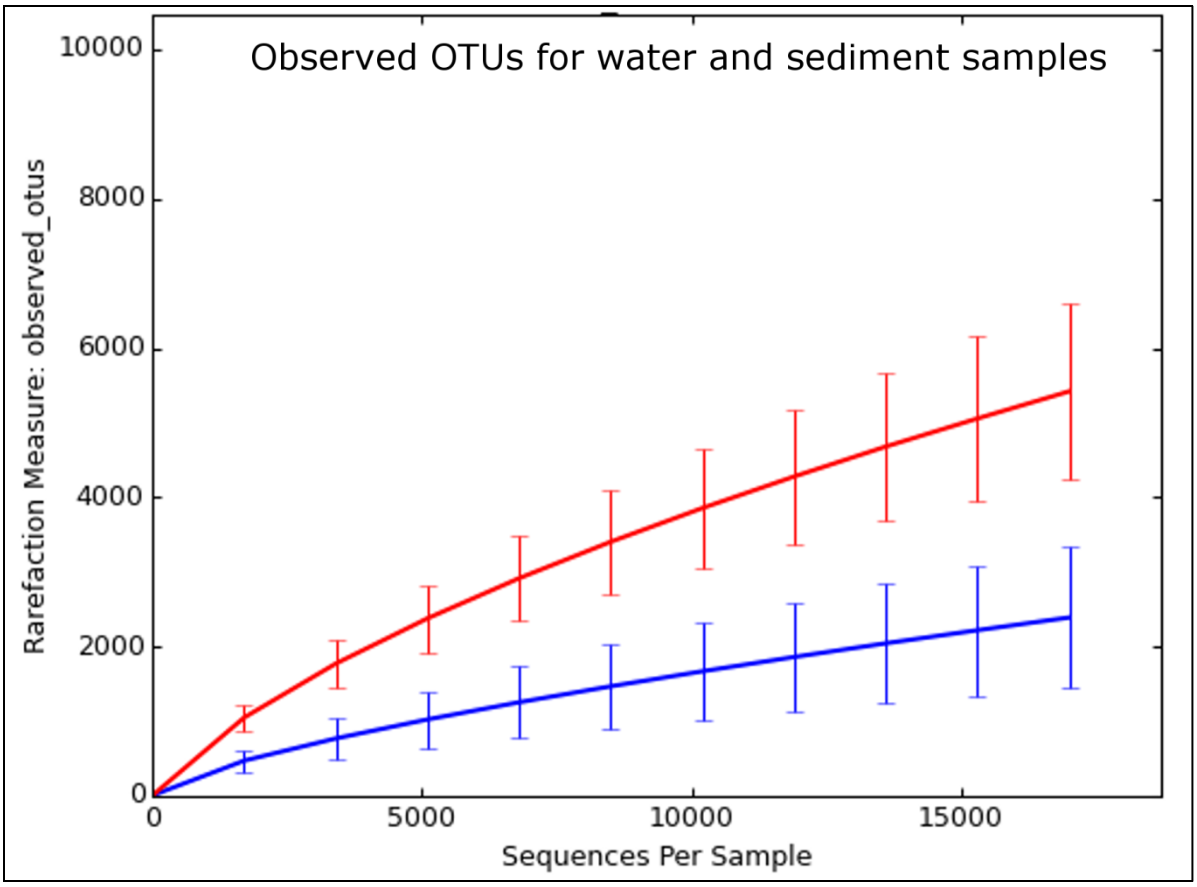

Figure S2 Legend: Rarefaction curves of observed OTUs up to 17,000 sequences for 277 water (blue) and 192 sediment (red) samples. The error bars mark the standard errors.
