# Supplementary material for: Spatial and Temporal Microbial Patterns in a Tropical Macrotidal Estuary Subject to Urbanization
Source: Front Microbiol. 2017 Jul 13;8:1313. doi: 10.3389/fmicb.2017.01313 (PMC5507994; doi:10.3389/fmicb.2017.01313)

Figure S3 PCO of water microbiota

Figure S3 Legend: Unconstrained principal coordinate ordination (PCO) plots based on the weighted Unifrac distance matrix of the water microbiota i.e. the OTU data of all water samples. **S3 A)** and **B)** show the same PCO for East Arm with **A)** labelled for sites and **B)** labelled for rounds of sampling. The same goes for **S3 C)** and **D)** for Shoal Bay. Both harbour areas show a clear separation along the first PCO axis of the microbiota impacted by effluent (red symbols on left) as compared to the control creeks (blue symbols on right). The microbiota in East Arm also showed a clear clustering according to season (**S3 B**) which was not evident for Shoal Bay (**S3 D**). The first two PCO axes of the East Arm microbiota explained 47.3 % of the data variance which compared to 44.2% for the Shoal Bay microbiota.

S3 A) East Arm – labelled for sites

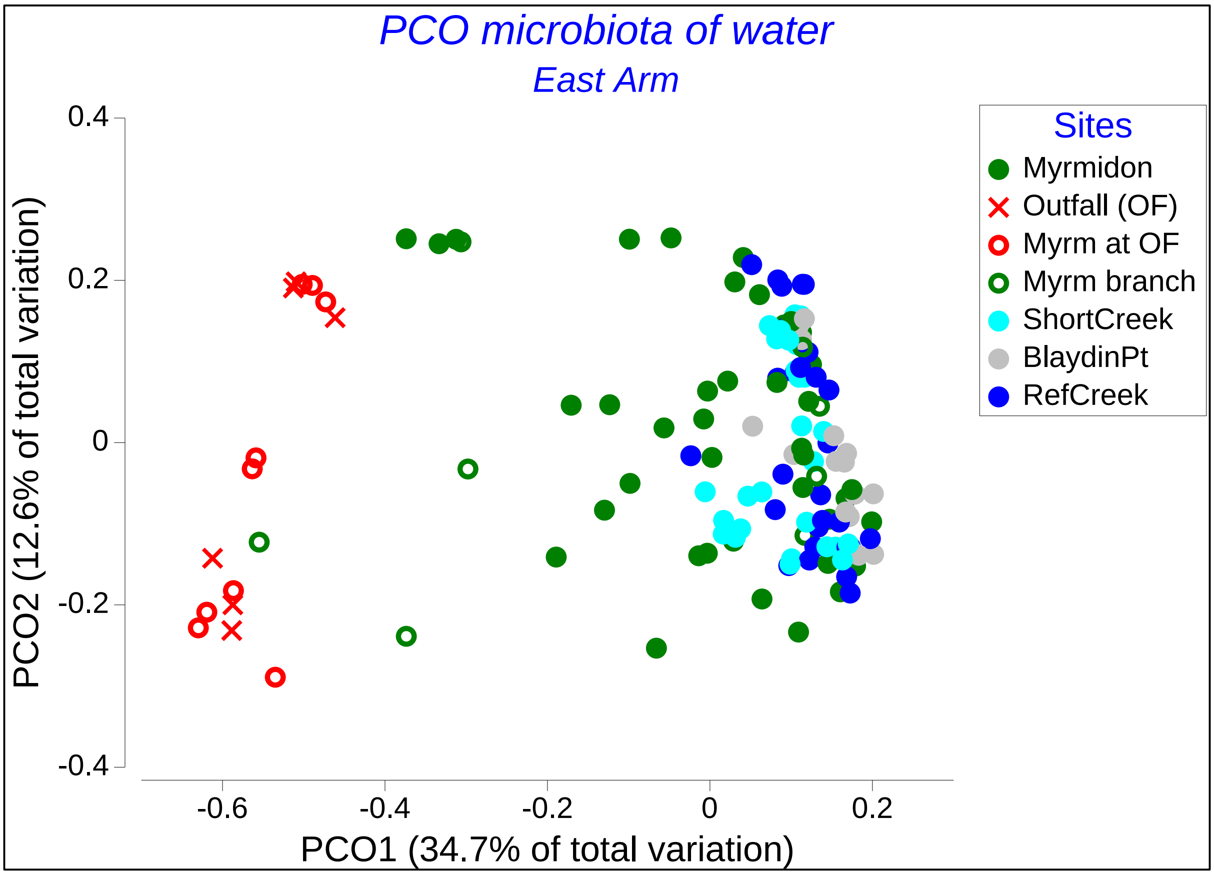

S3 B) East Arm – labelled for sampling round

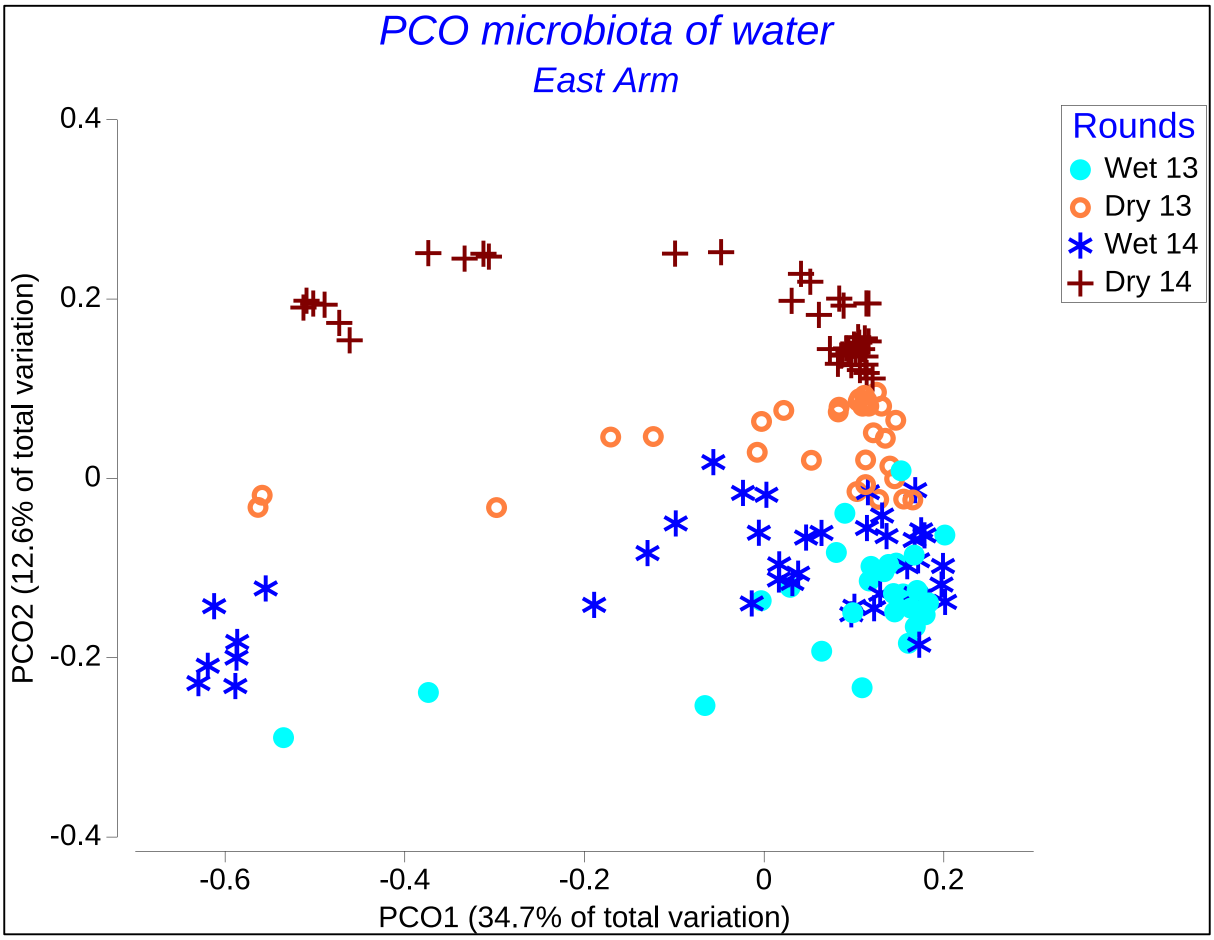

S3 C) Shoal Bay – labelled for sites

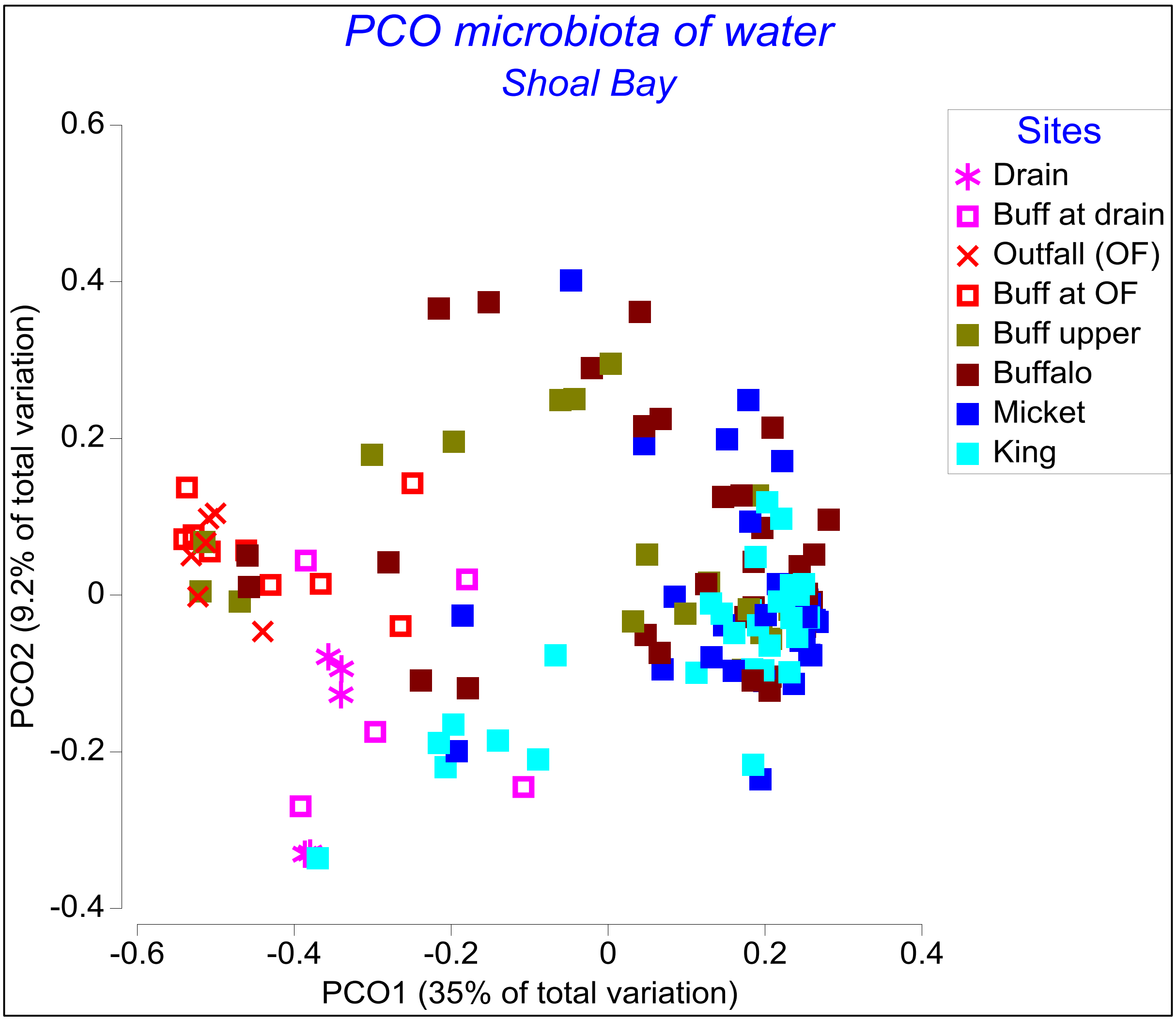

S3 D) Shoal Bay – labelled for sampling round

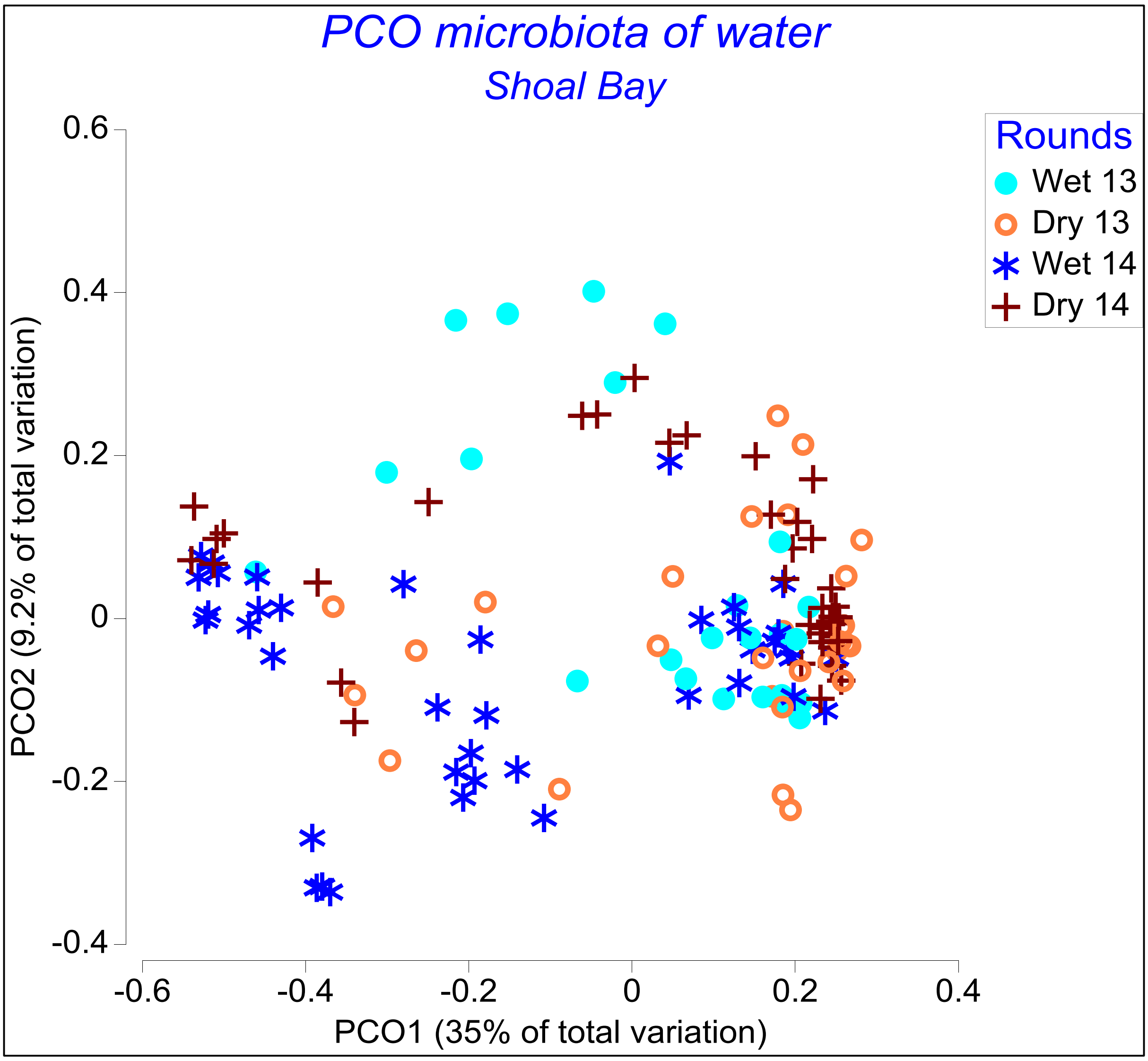

Supplement: Supplementary file 3 [file Image3.PDF]
