# Supplementary material for: Spatial and Temporal Microbial Patterns in a Tropical Macrotidal Estuary Subject to Urbanization
Source: Front Microbiol. 2017 Jul 13;8:1313. doi: 10.3389/fmicb.2017.01313 (PMC5507994; doi:10.3389/fmicb.2017.01313)

Figure S4: PCO of water nutrients

Figure S4 Legend: PCO based on Euclidean distance matrix of the log-transformed and normalised abiotic factors. For East Arm (S4 A), the first two PCO axes explained 76% of the variance in the abiotic factors and for Shoal Bay (S4 B) 74%.

S4 A) East Arm

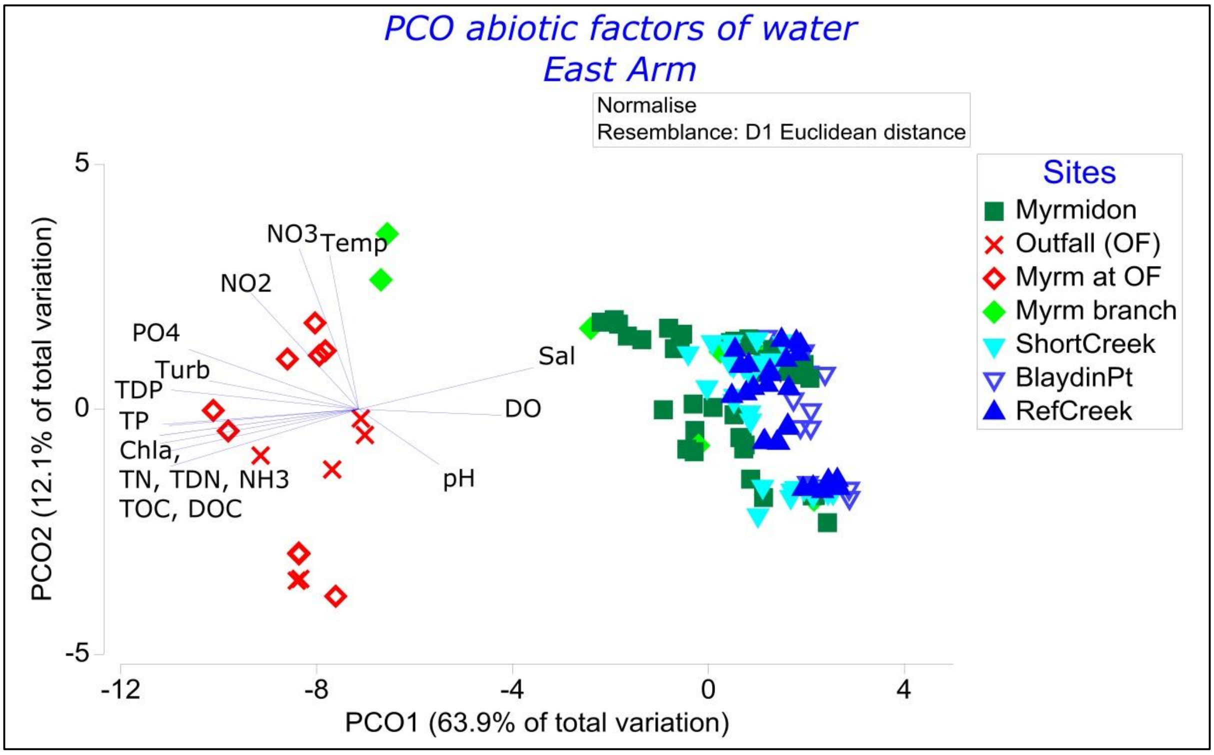

S4 B) Shoal Bay

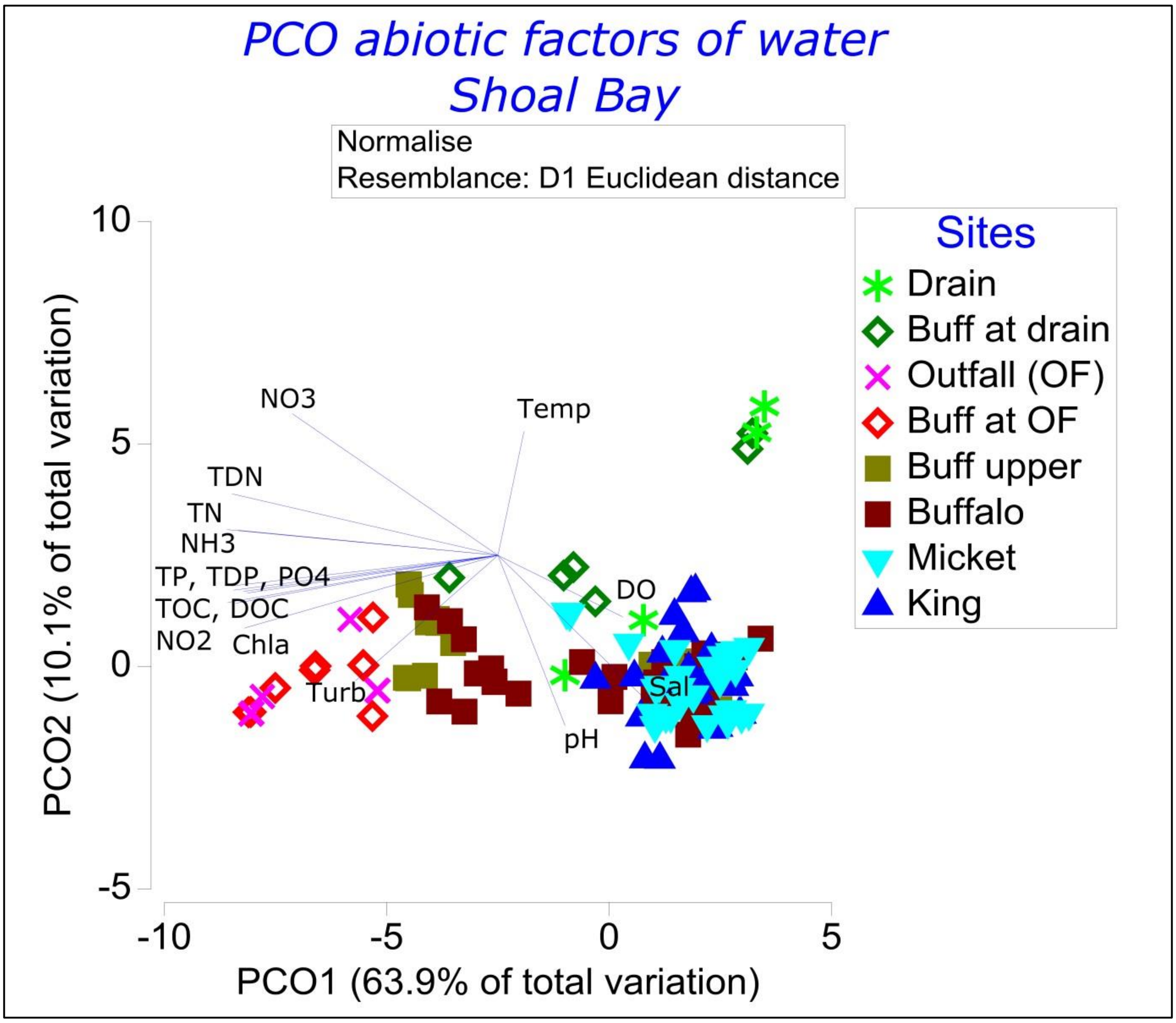

Supplement: Supplementary file 4 [file Image4.PDF]
