# Supplementary material for: Spatial and Temporal Microbial Patterns in a Tropical Macrotidal Estuary Subject to Urbanization
Source: Front Microbiol. 2017 Jul 13;8:1313. doi: 10.3389/fmicb.2017.01313 (PMC5507994; doi:10.3389/fmicb.2017.01313)

Figure S5: dbRDA of water microbiota

Figure S5 Legend: Distance-based redundancy analysis (dbRDA) plots of **A)** East Arm and **B)** Shoal Bay water microbiota. The dbRDA visualizes the distance-based linear model which associates the microbiota with abiotic factors (see Table 3). The base eigenvectors show the direction and strength of the factors which most explained the variance of the microbiota. The vectors labelled in colour indicate the two top drivers for each model (i.e. temperature and NH_3 for **A)** and NO_2 and salinity for **B)**). The size of the half-bubbles in the top graphs are proportional to the concentration of the corresponding factor in the samples. The bottom graph shows the same dbRDA with samples labelled according to their site of collection. The distinct cluster of the dry season 2014 microbiota in East Arm is shown in the bottom graph of **A)** and was mainly associated with lower temperature, higher dissolved oxygen and higher salinity. No such round-specific clustering was evident for Shoal Bay **(B)**. The corresponding unconstrained PCOs of A) and B) are shown in the Supplementary Figure S3.

S5 A) East Arm

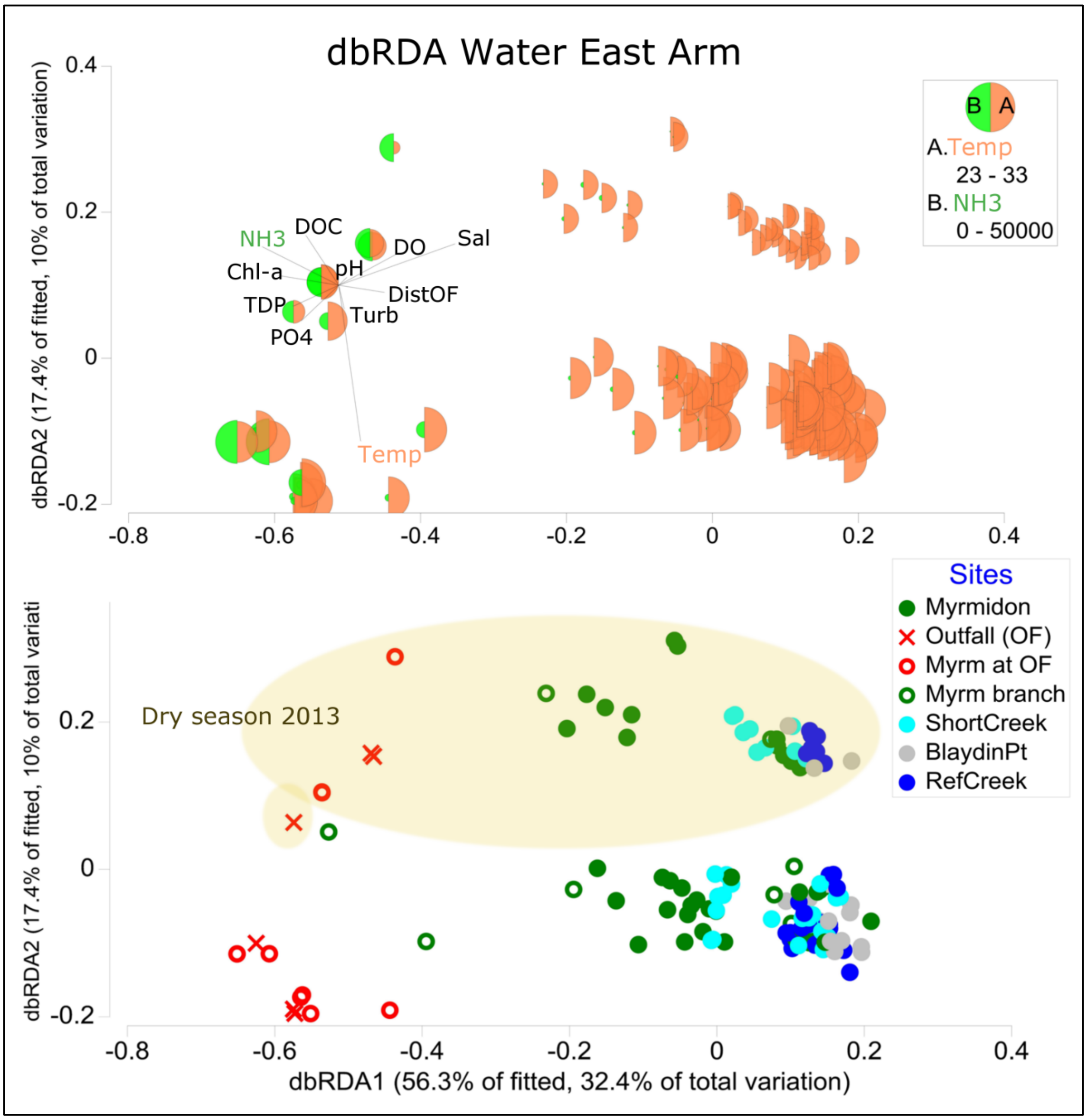
dbRDA Water East Arm
dbRDA2 (17.4% of fitted, 10% of total variation)
NH3
DOC
DO
Sal
Chl-a
pH
DistOF
TDP
Turb
PO4
Temp
B A
A. Temp
23 - 33
B. NH3
0 - 50000
Sites
Myrmidon
Outfall (OF)
Myrm at OF
Myrm branch
ShortCreek
BlaydinPt
RefCreek
Dry season 2013
dbRDA2 (17.4% of fitted, 10% of total variati
dbRDA1 (56.3% of fitted, 32.4% of total variation)

S5 B) Shoal Bay

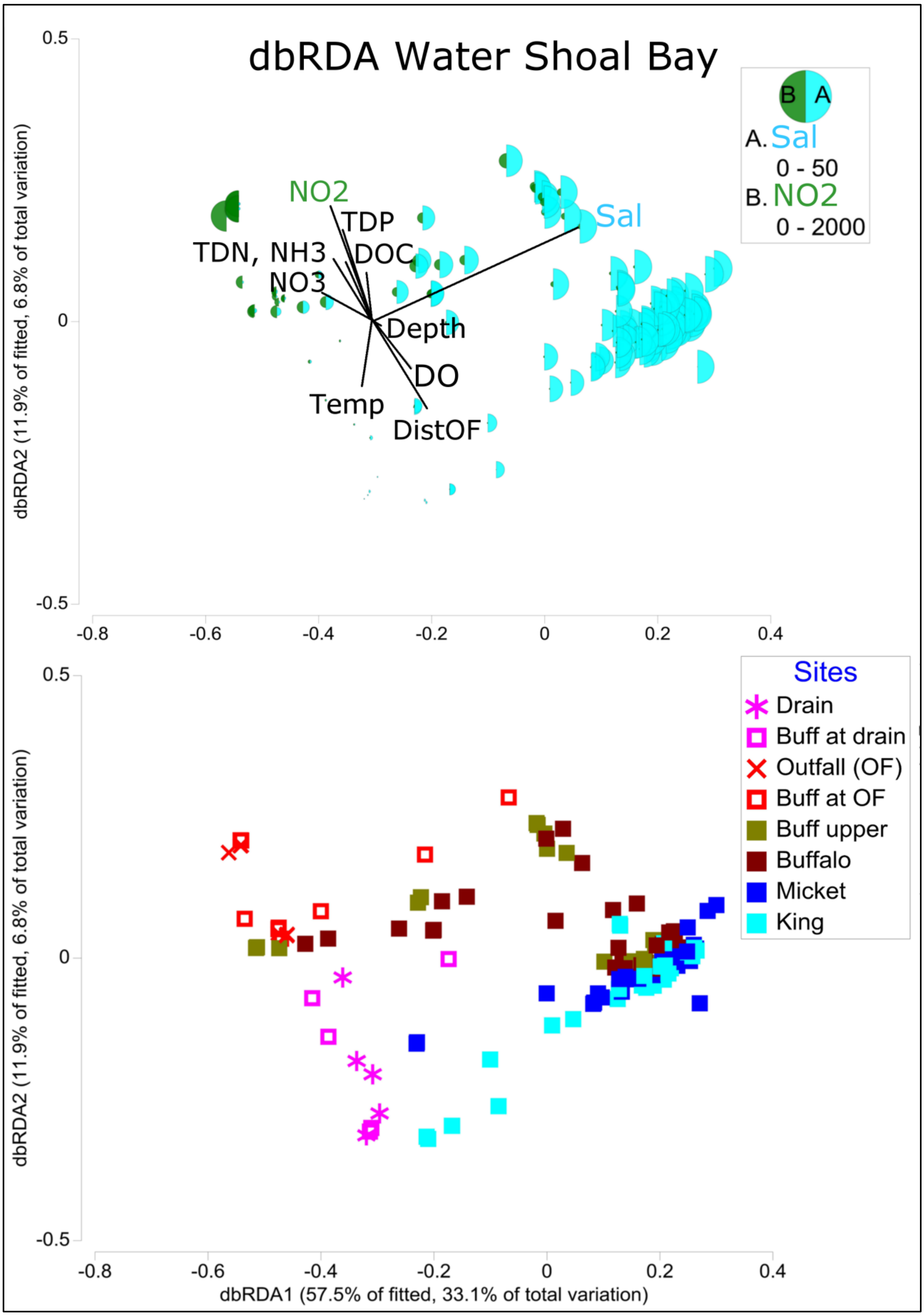

Supplement: Supplementary file 5 [file Image5.PDF]
