# Supplementary material for: Spatial and Temporal Microbial Patterns in a Tropical Macrotidal Estuary Subject to Urbanization
Source: Front Microbiol. 2017 Jul 13;8:1313. doi: 10.3389/fmicb.2017.01313 (PMC5507994; doi:10.3389/fmicb.2017.01313)
Supplement: Supplementary file 6 [file Image6.PDF]

Figure S6: PCNM analysis

Eigenvector-based spatial model of the neap water microbiota in Shoal Bay (**A to C**) and East Arm (**D**) using Principal Coordinates of Neighbour Matrices (PCNM) eigenfunctions.

This method produces orthogonal spatial eigenvectors for neighbouring sampling sites exhibiting positive spatial correlation (based on Moran's I). The threshold for neighbouring sites was set using the default minimum spanning tree method to ensure that all sites were connected. These spatial eigenvectors were then used as explanatory variables in a RDA analysis to test their association with the microbial community structure.

Circles in Figure S6 represent the sampling sites (location based on distances across water between sites). The stars indicate the effluent outfall sites at Buffalo and Myrmidon Creeks and the hashtag the urban runoff at Buffalo Creek. Large circles indicate large positive (black) or negative (white) eigenvalues of those spatial eigenvectors which were significantly associated with the microbial variation (P=0.001 for all displayed). These were three eigenvectors for Shoal Bay (**A-C**) and one for East Arm (**D**). These eigenvectors reflect spatial patterns which explained significant parts of the microbial variance as outlined by the R^2 which was adjusted for the number of explanatory variables.

The pattern in **A)** shows a linear trend for Shoal Bay along the x-axis with the microbiota being more similar to each other in King Creek and upper Micket Creek and most different to the microbiota in Buffalo Creek. **B)** shows a linear trend along the y-axis with Micket Creek microbiota differing from King Creek and upper Buffalo Creek **C)** shows a non-linear pattern with the urban runoff and most upstream Micket Creek correlated and **D)** shows a linear trend for East Arm with the microbiota in Myrmidon more similar and opposed to the microbiota in the reference creeks.

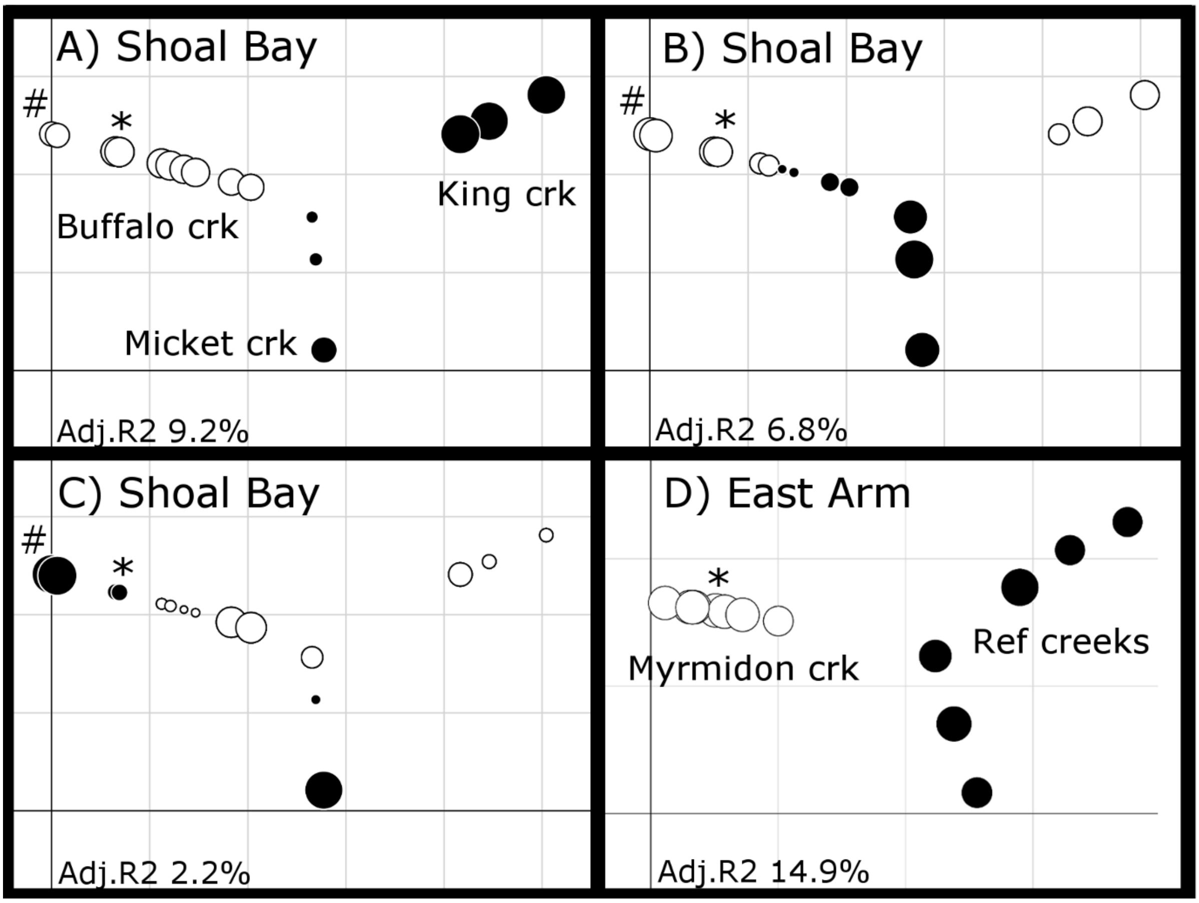
A) Shoal Bay
#
*
Buffalo crk
King crk
Micket crk
Adj.R2 9.2%
B) Shoal Bay
#
*
Adj.R2 6.8%
C) Shoal Bay
#
*
Adj.R2 2.2%
D) East Arm
*
Myrmidon crk
Ref creeks
Adj.R2 14.9%
