# Supplementary figures and images for: Spatial and Temporal Microbial Patterns in a Tropical Macrotidal Estuary Subject to Urbanization
Source: Front Microbiol. 2017 Jul 13;8:1313. doi: 10.3389/fmicb.2017.01313 (PMC5507994; doi:10.3389/fmicb.2017.01313)

Figure S7: Phyla plot of sediment and water phyla

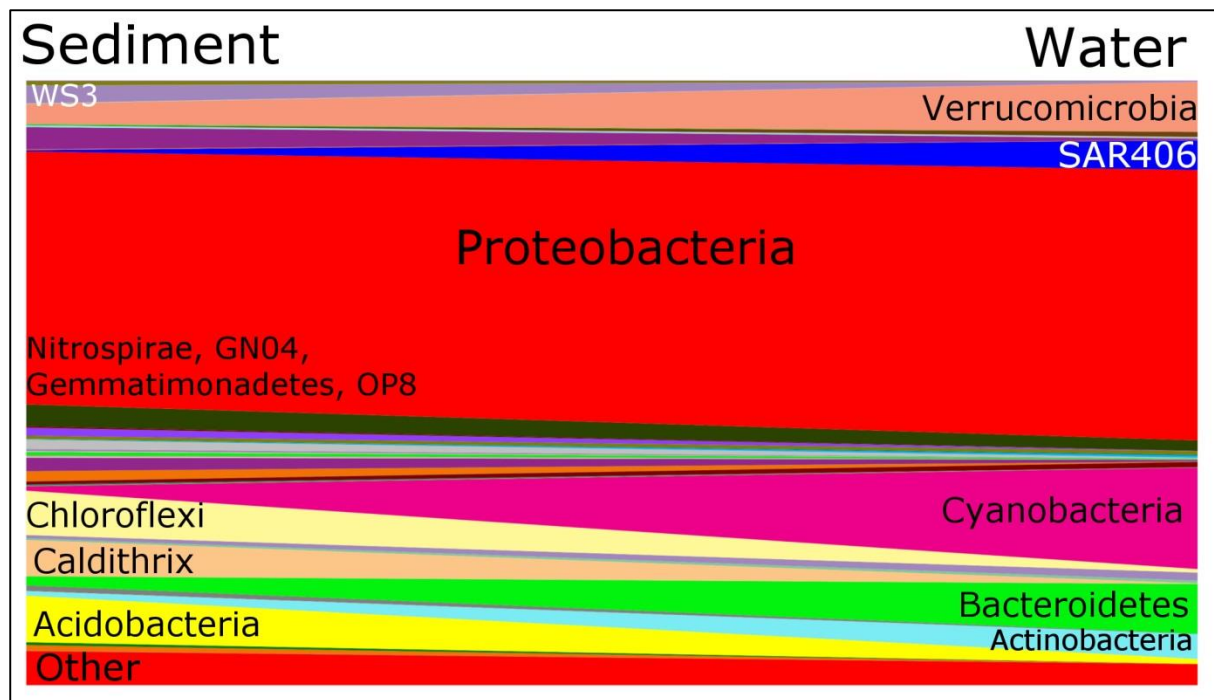

Supplement: Supplementary file 7 [file Image7.PDF]
