# Supplementary material for: Spatial and Temporal Microbial Patterns in a Tropical Macrotidal Estuary Subject to Urbanization
Source: Front Microbiol. 2017 Jul 13;8:1313. doi: 10.3389/fmicb.2017.01313 (PMC5507994; doi:10.3389/fmicb.2017.01313)
Supplement: Supplementary file 8 [file Image8.PDF]

Figure S8 Simpson Diversity

S8 A) Water

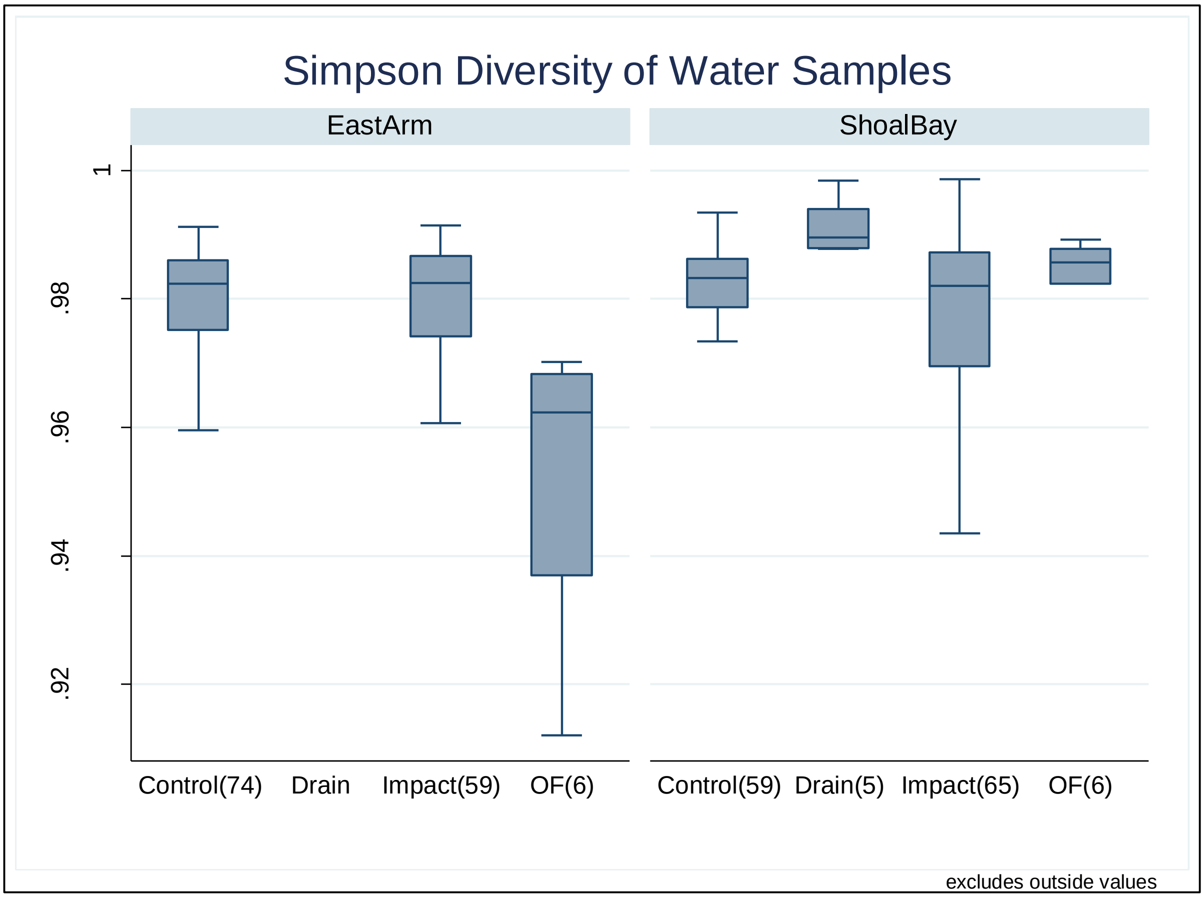

Figure S8 A) Legend: Simpson diversity of water samples from East Arm and Shoal Bay. Numbers in brackets indicate the number of samples

S8 B) Sediment

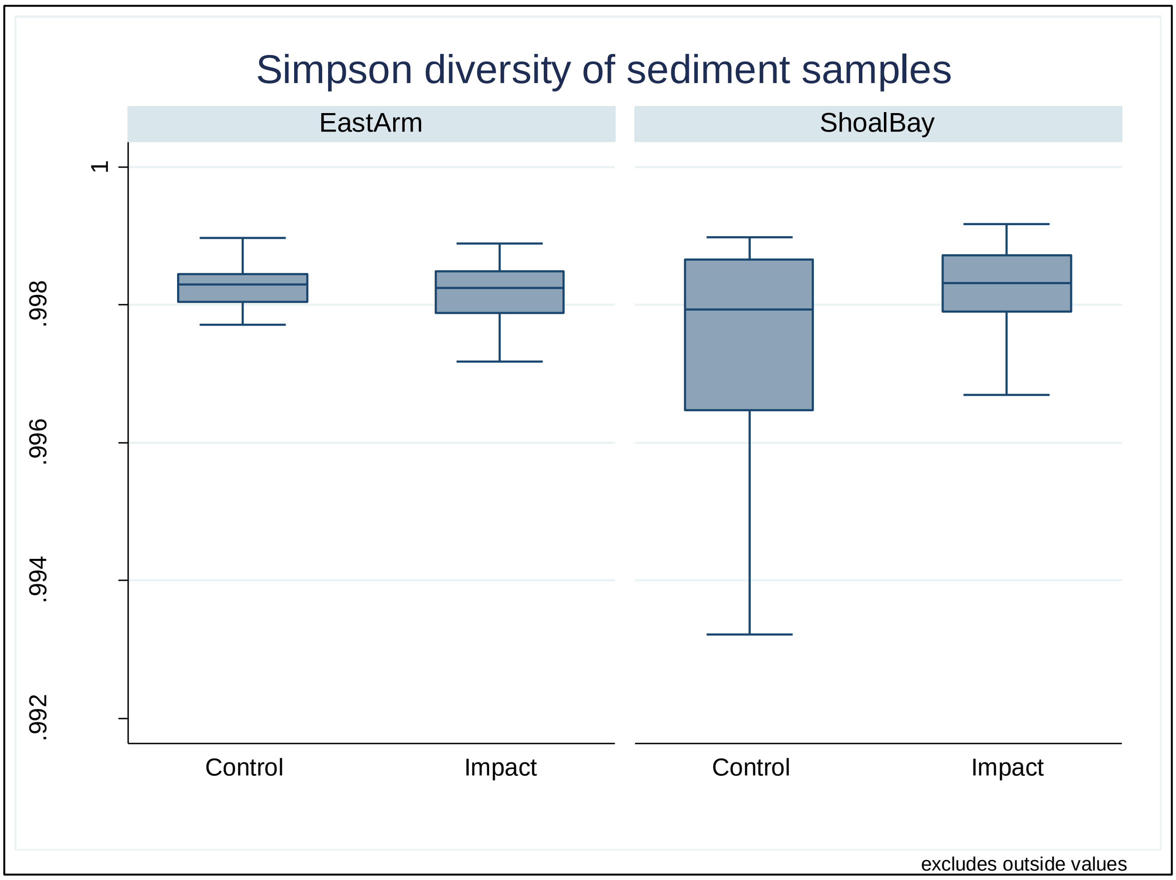

Figure S8 B) Legend: Simpson diversity of sediment samples from East Arm and Shoal Bay. For East Arm, there were 39 sediment samples from control creeks and 48 samples from impacted Myrmidon creek while for Shoal Bay, there were 42 control and 54 impacted sediment samples.
