# Supplementary material for: Spatial and Temporal Microbial Patterns in a Tropical Macrotidal Estuary Subject to Urbanization
Source: Front Microbiol. 2017 Jul 13;8:1313. doi: 10.3389/fmicb.2017.01313 (PMC5507994; doi:10.3389/fmicb.2017.01313)

Figure S9: PCO of sediment samples averaged by site

The weighted unifrac dissimilarity matrix was averaged by site with trajectories for samples along Buffalo creek (blue) and Myrmidon creek (orange dashed). No clear path is evident for neither harbour area.

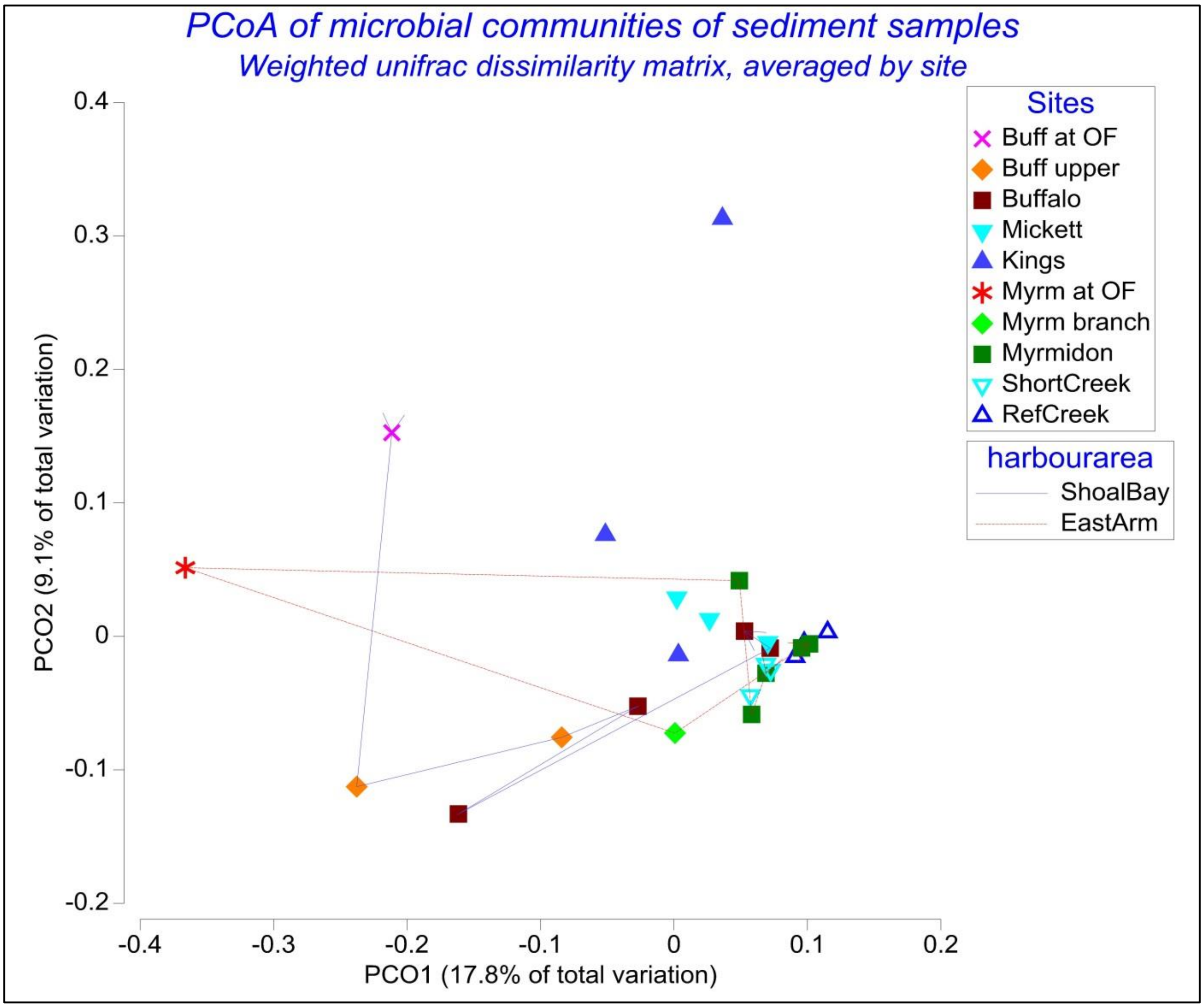

Supplement: Supplementary file 9 [file Image9.PDF]
