# Supplementary material for: Spatial and Temporal Microbial Patterns in a Tropical Macrotidal Estuary Subject to Urbanization
Source: Front Microbiol. 2017 Jul 13;8:1313. doi: 10.3389/fmicb.2017.01313 (PMC5507994; doi:10.3389/fmicb.2017.01313)

Figure S10: PCO & CAP of sediment

Figure S10 A)-B) East Arm

S10 A)-B) Legend: PCO (A) and CAP constrained for creeks (B) for sediment samples from East Arm. For the CAP, 52 of 86 PCO axes were used to maximize the correct classification rate.

A)

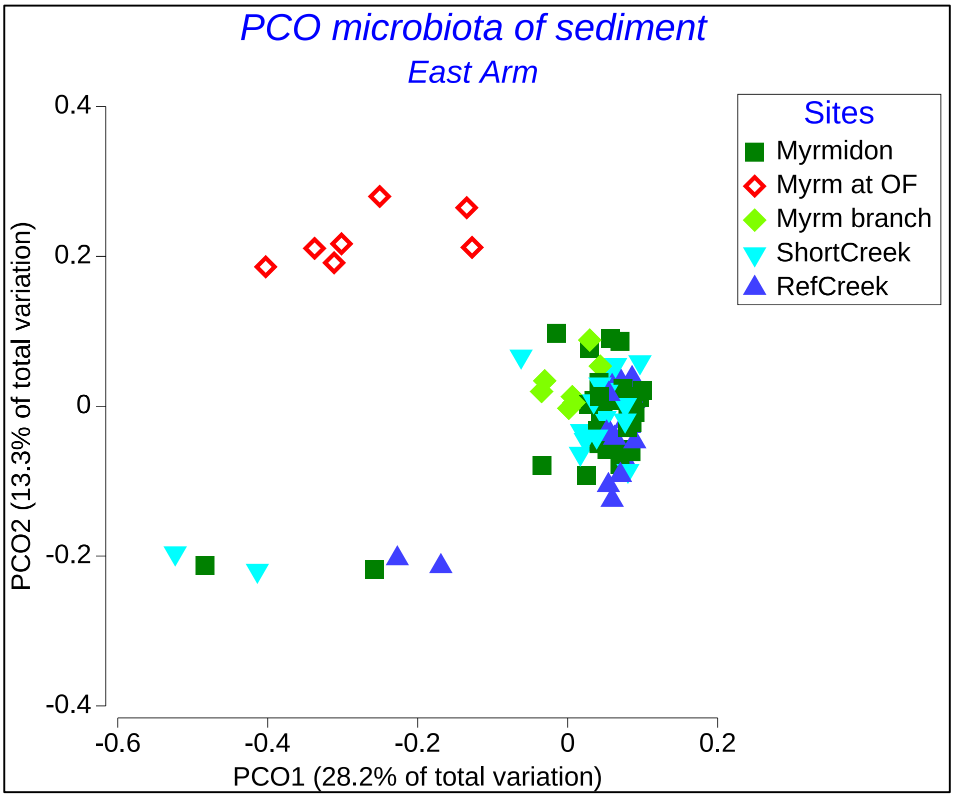

B)

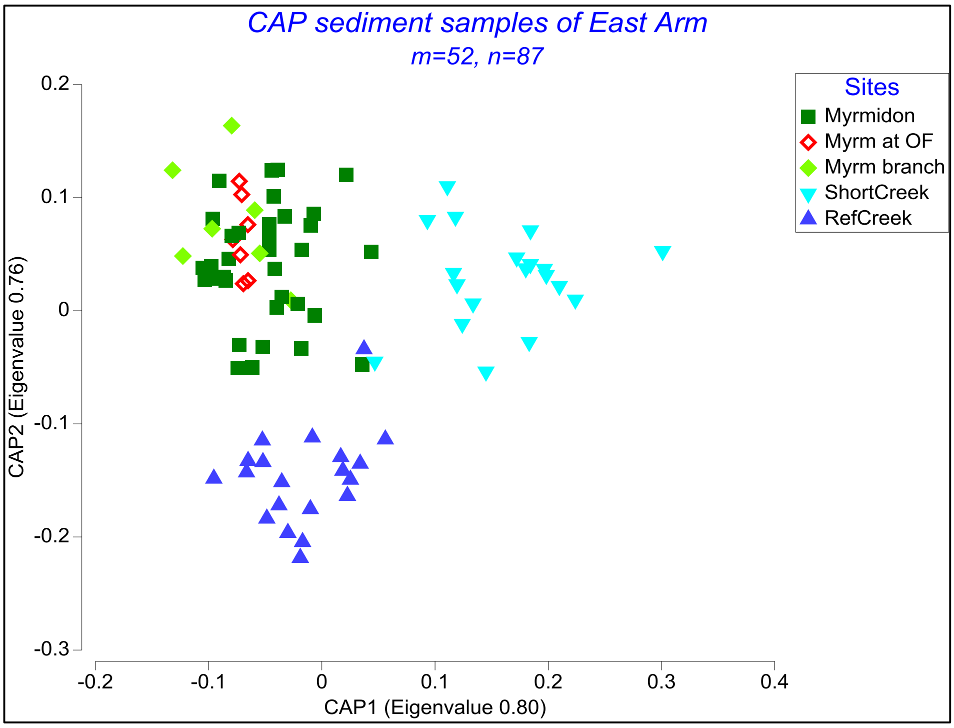

Figure S10 C)-D) Shoal Bay

S10 C)-D) Legend: PCO (C) and CAP constrained for creeks (D) for sediment samples from Shoal Bay. For the CAP, 79 of 95 PCO axes were used to maximize the correct classification rate.

A)

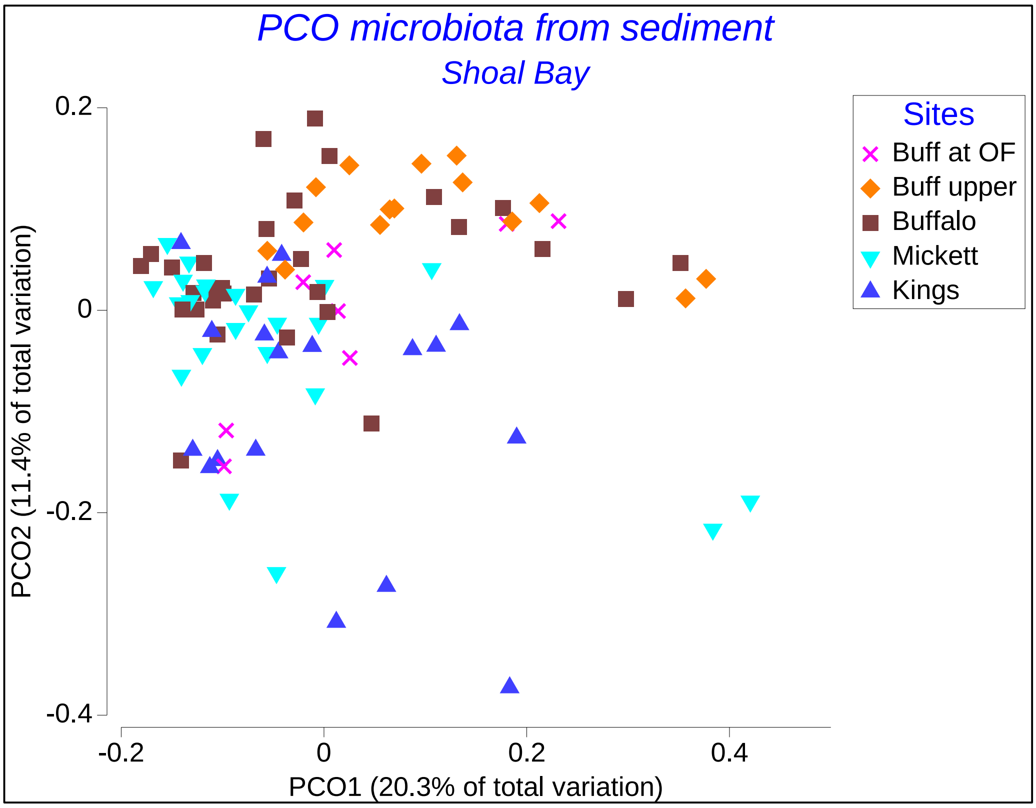

B)

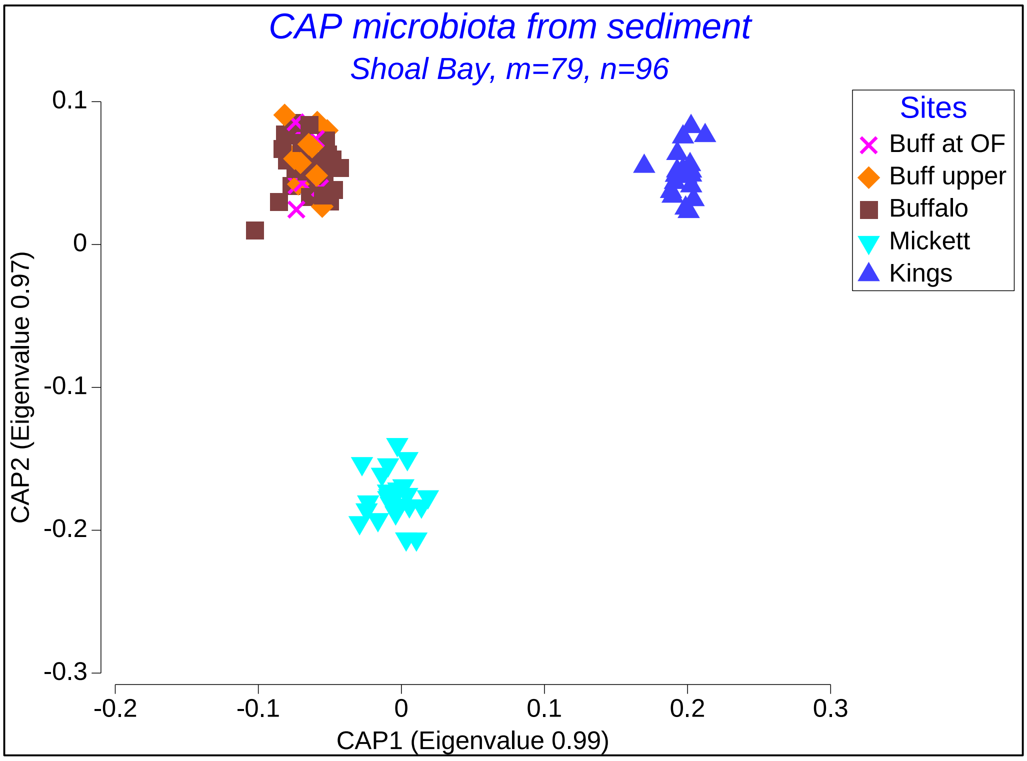

Supplement: Supplementary file 10 [file Image10.PDF]
