# Supplementary material for: Spatial and Temporal Microbial Patterns in a Tropical Macrotidal Estuary Subject to Urbanization
Source: Front Microbiol. 2017 Jul 13;8:1313. doi: 10.3389/fmicb.2017.01313 (PMC5507994; doi:10.3389/fmicb.2017.01313)
Supplement: Supplementary file 11 [file Image11.PDF]

Figure S11: East Arm sediment microbiota & abiotic factors

S11 A)-C) Legend: A) PCO of physicochemical factors of sediment of East Arm and **B)** dbRDA based on a distance linear model using stepwise selection and the AIC criterion to choose the best model with the weighted Unifrac distance matrix of the sediment microbiota as outcome and the log transformed and normalized abiotic factors as independent factors. As ORP proved an important factor but was only measured in the second year of the study, the below analysis is based on the second year of the study. The first two dbRDA axes explained 42.7% of the total sediment microbiota. **C)** CCA of the raw OTU data of the sediment microbiota (OTUs which occurred in less than 10 samples were excluded) and log transformed and normalized abiotic factors. The most parsimonious model was chosen based on the ordistep procedure. The first two CCA axes captured 27.8% of the sediment microbiota variance. Four vectors were not displayed due to collinearity with the Mn vector (Pb) or the Cu vector (P, TKN and Zn) with variance inflation factors of 5.8 (Pb) to 70 for Zn. Both, OTU and sample scores were scaled symmetrically by the square root of the CCA eigenvalues.

A)

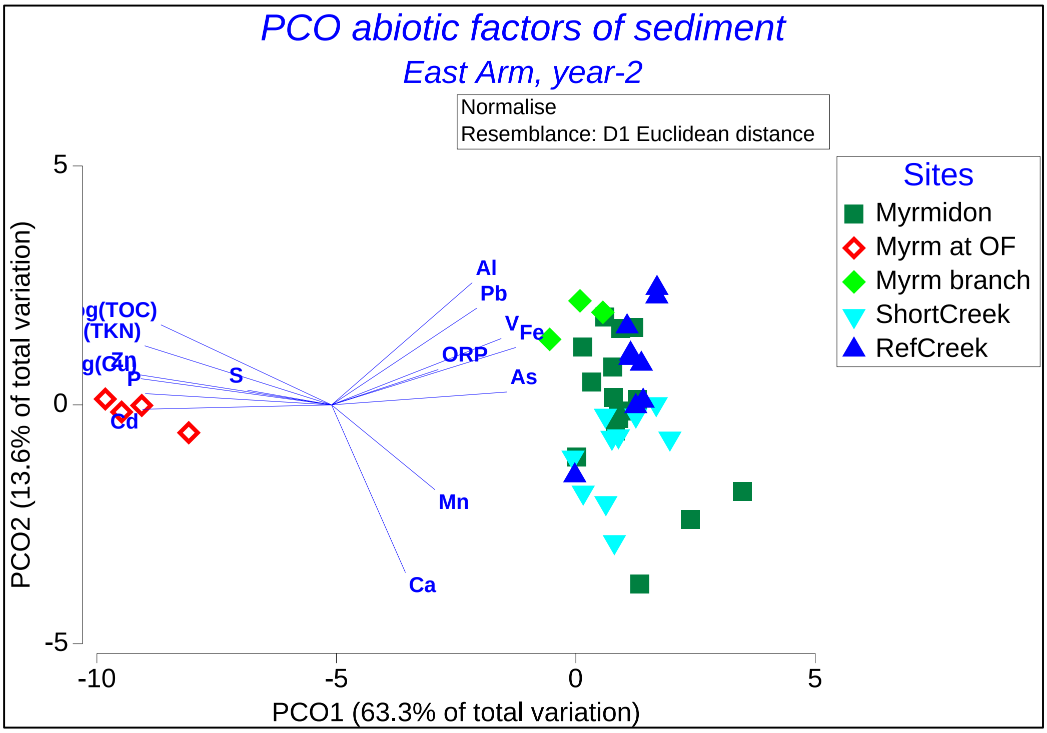

B)

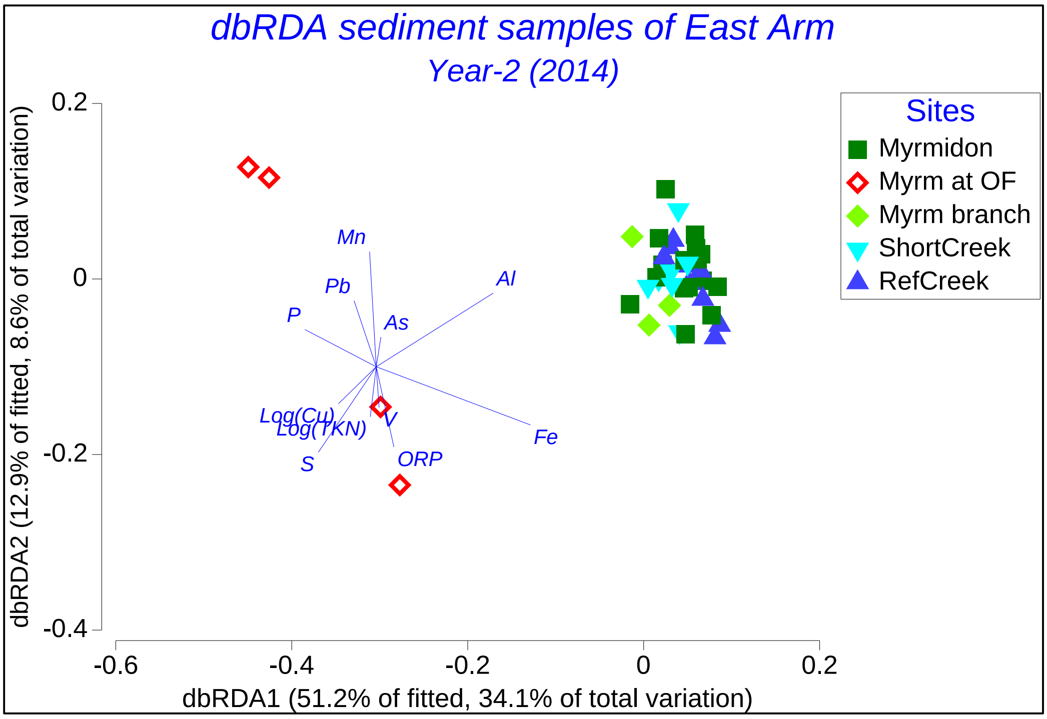

dbRDA sediment samples of East Arm
Year-2 (2014)
Sites
Myrmidon
Myrm at OF
Myrm branch
ShortCreek
RefCreek
Mn
Pb
Al
P
As
Log(Cu)
Log(TKN)
V
Fe
S
ORP
0.2
0
-0.2
-0.4
-0.6
-0.4
-0.2
0
0.2
dbRDA2 (12.9% of fitted, 8.6% of total variation)
dbRDA1 (51.2% of fitted, 34.1% of total variation)

C)

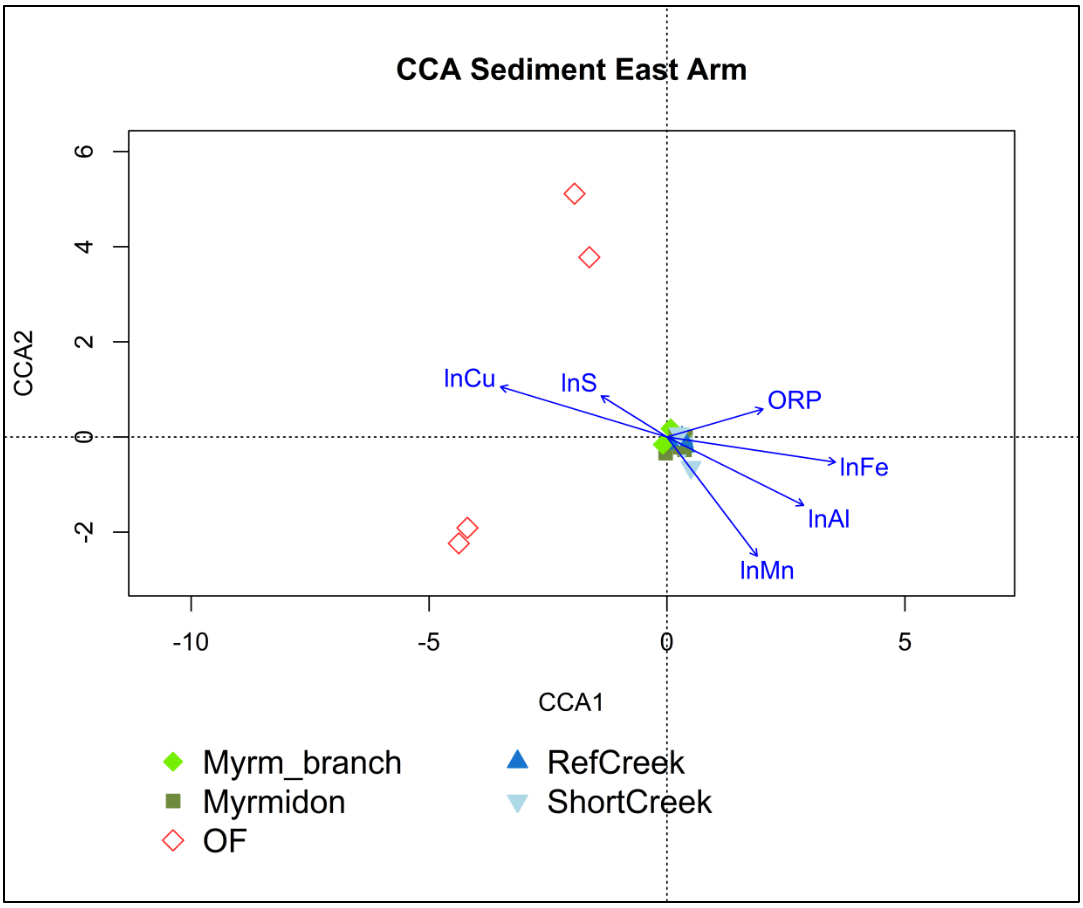

CCA Sediment East Arm
CCA2
CCA1
6
4
2
0
-2
-10
-5
0
5
lnCu
lnS
ORP
lnFe
lnAl
lnMn
Myrm_branch
Myrmidon
OF
RefCreek
ShortCreek
