# Supplementary material for: Spatial and Temporal Microbial Patterns in a Tropical Macrotidal Estuary Subject to Urbanization
Source: Front Microbiol. 2017 Jul 13;8:1313. doi: 10.3389/fmicb.2017.01313 (PMC5507994; doi:10.3389/fmicb.2017.01313)
Supplement: Supplementary file 12 [file Image12.PDF]

Figure S12: Shoal Bay sediment microbiota & abiotic factors

S12 A)-C) Legend: A) PCO of physicochemical factors of sediment of Shoal Bay and **B)** dbRDA based on a distance linear model using stepwise selection and the AIC criterion to choose the best model with the weighted Unifrac distance matrix of the sediment microbiota as outcome and the log transformed and normalized abiotic factors as independent factors. As ORP proved an important factor but was only measured in the second year of the study, the below analysis is based on the second year of the study. The first two dbRDA axes explained 26.4% of the total sediment microbiota. **C)** CCA of the raw OTU data of the sediment microbiota (OTUs which occurred in less than 10 samples were excluded) and log transformed and normalized abiotic factors. The most parsimonious model was chosen based on the ordistep procedure. The first two CCA axes only captured 15.5% of the sediment microbiota variance. The Pb vector was not displayed due to collinearity with the Cu vector with a variance inflation factor of 16. Both, OTU and sample scores were scaled symmetrically by the square root of the CCA eigenvalues.

A)

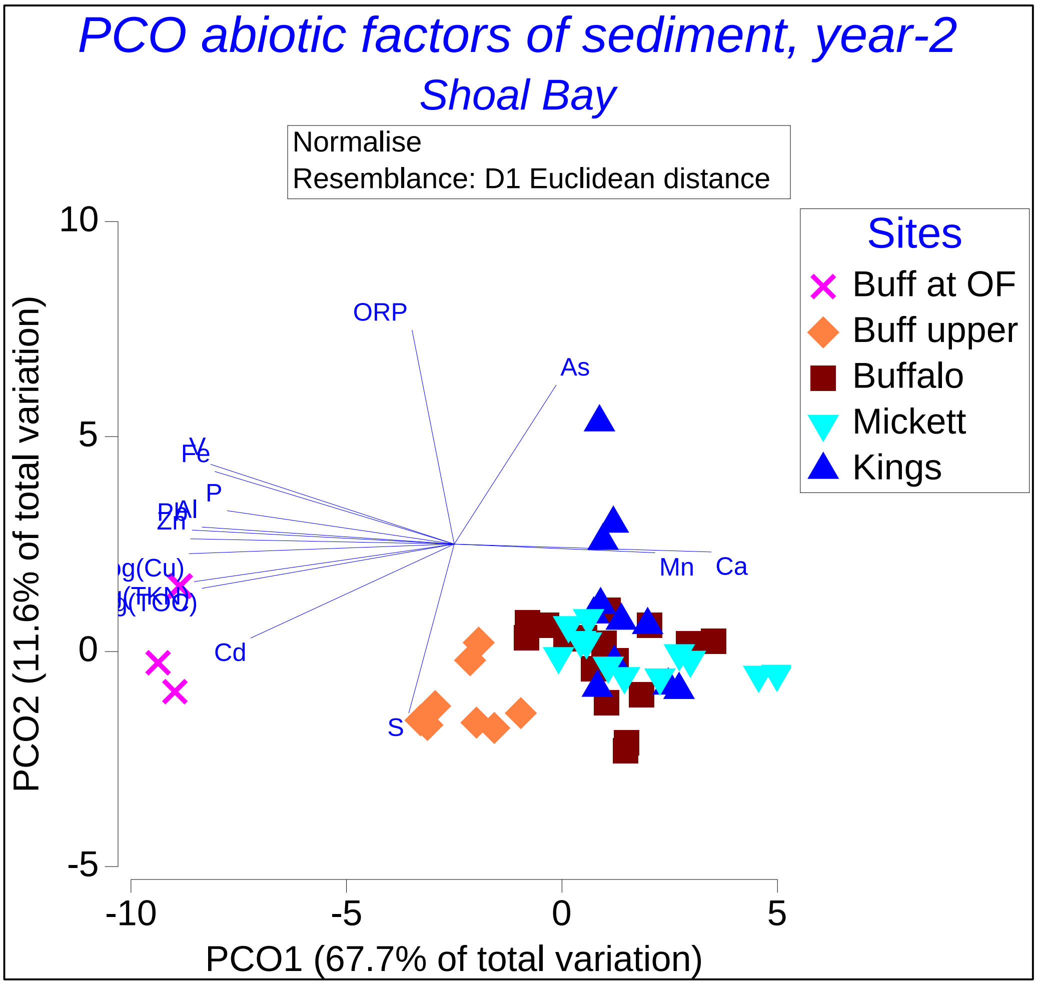

B)

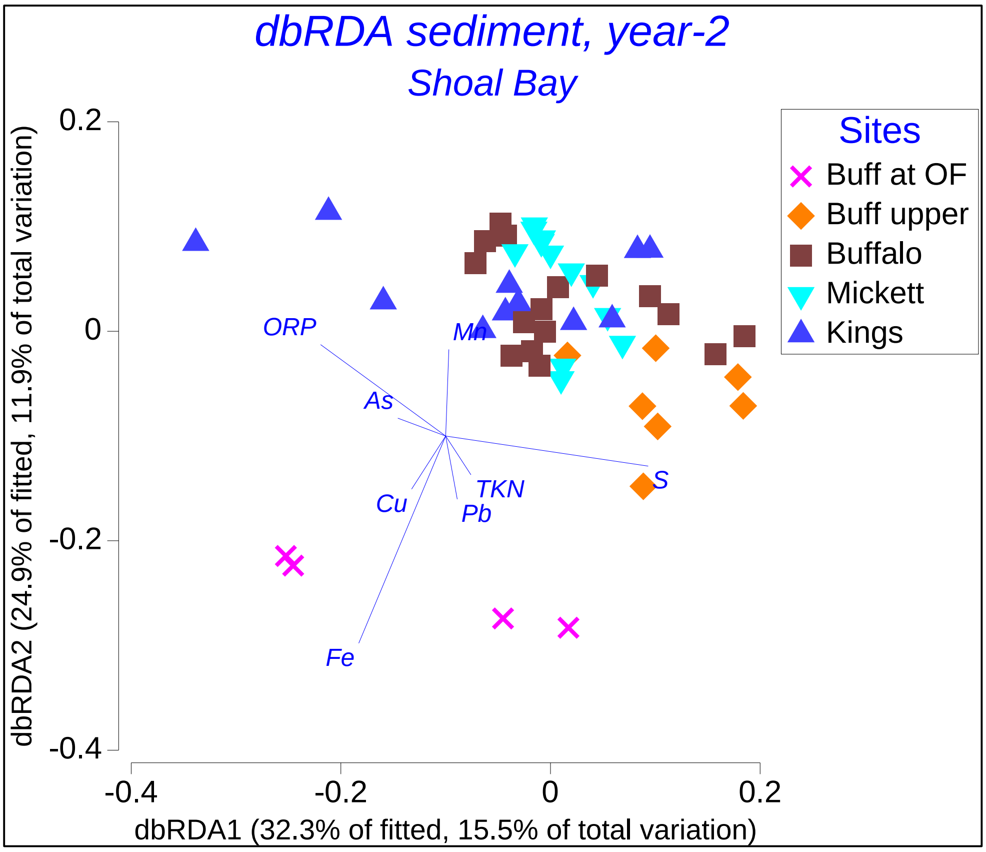
dbRDA sediment, year-2
Shoal Bay
Sites
Buff at OF
Buff upper
Buffalo
Mickett
Kings
ORP
Mn
As
S
TKN
Cu
Pb
Fe
0.2
0
-0.2
-0.4
-0.4
-0.2
0
0.2
dbRDA2 (24.9% of fitted, 11.9% of total variation)
dbRDA1 (32.3% of fitted, 15.5% of total variation)

C)

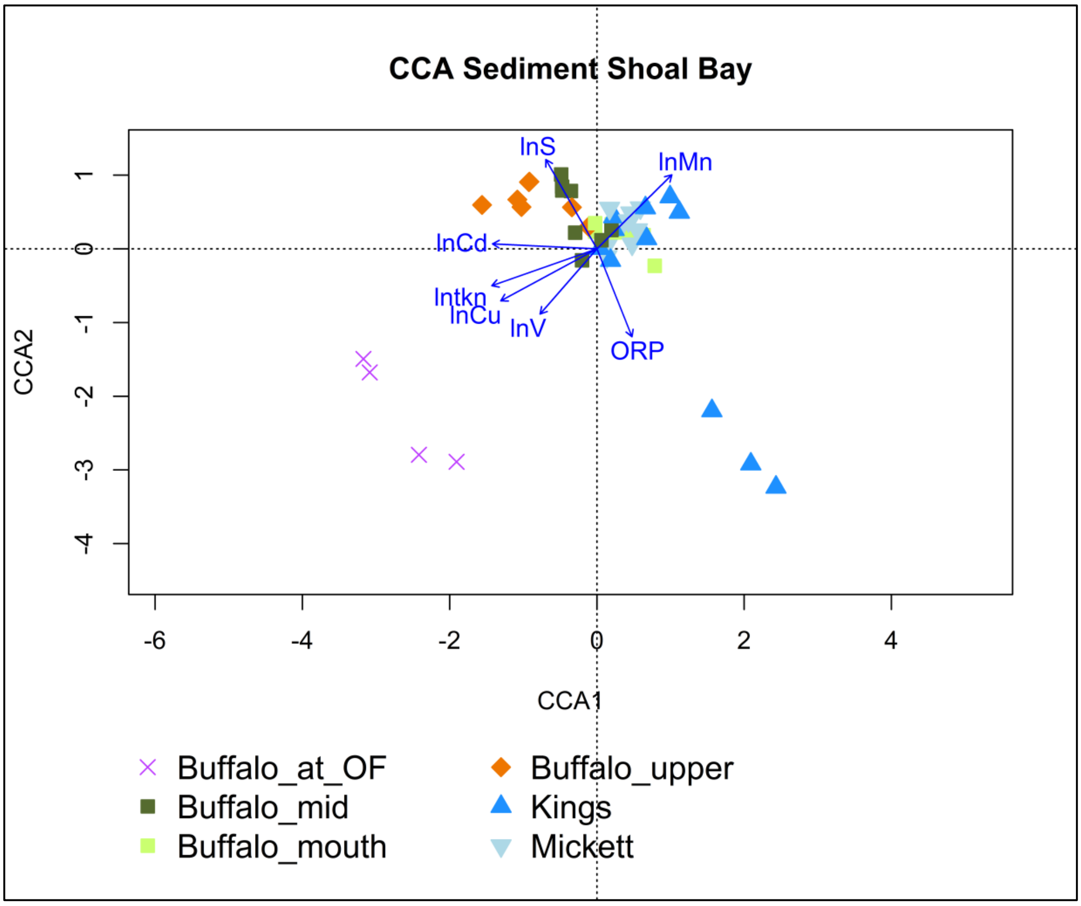
CCA Sediment Shoal Bay
lnS
lnMn
lnCd
lntkn
lnCu
lnV
ORP
1
0
-1
-2
-3
-4
-6
-4
-2
0
2
4
CCA2
CCA1
Buffalo_at_OF
Buffalo_mid
Buffalo_mouth
Buffalo_upper
Kings
Mickett
