# Supplementary material for: Spatial and Temporal Microbial Patterns in a Tropical Macrotidal Estuary Subject to Urbanization
Source: Front Microbiol. 2017 Jul 13;8:1313. doi: 10.3389/fmicb.2017.01313 (PMC5507994; doi:10.3389/fmicb.2017.01313)
Supplement: Supplementary file 13 [file Table1.DOCX]

# S1 Choice of sequencing pipeline and subsampling

The MOTHUR ([Schloss et al., 2009](#_ENREF_6)) (v 1.34.1), UPARSE ([Edgar, 2013](#_ENREF_3)) (64-bit v8), QIIME ([Caporaso et al., 2010](#_ENREF_1)) (v 1.9.0) closed and open reference MiSeq SOP pipelines were run in a linux environment on the high performance computer at Charles Darwin University using the default parameters unless otherwise stated.

A uchime chimera-removal step was included in all pipelines ([Edgar et al., 2011](#_ENREF_4)), the minimum identity for an OTU cluster was 97% for all pipelines and singleton OTUs were discarded. The Greengenes database was used for taxonomy assignment (release May 2013)([McDonald et al., 2012](#_ENREF_5)).

The suitability of these pipelines for our data (277 estuarine water and 192 sediment samples) was compared using the criteria in Table S1 and Figure S1. Subsampling to 17,000 sequences vs no subsampling were also compared as well as different distance matrices i.e. the Bray Curtis (on raw OTU data) vs. weighted Unifrac distance matrix (Table S1)(Figure S1).

Macrotidal tropical mangrove estuaries are a poorly characterised environment and accordingly, 44% of reads were discarded with the QIIME closed reference pipeline due to no taxonomic assignment using the Greengenes database (Table S1). Therefore, this pipeline was not followed further.

While the total number of OTUs was 8.2 times higher for the QIIME open reference pipeline compared to the UPARSE pipeline, their weighted Unifrac distance matrices were still highly correlated based on rank dissimilarities with a Spearman correlation of 0.94 (Figure S1). Both pipelines showed a similar explained variance in an unconstrained PCO (Table S1) with the weighted Unifrac and subsampling to 17,000 sequences showing the highest explained variance (Table S1).

The QIIME open reference was chosen over the UPARSE pipeline as its weighted Unifrac distance matrix was a good representative of the other tested approaches (Figure S1) and the PCO of its weighted Unifrac matrix showed a slightly higher explained variance (Table S1).

Subsampling to 17,000 sequences was performed with reasons outlined in the results of the main text.

The weighted Unifrac distance measure was chosen as it was less susceptible to change upon subsampling showing a correlation above 0.99 between the distance matrices based on all or subsampled sequences (Figure S1) . Furthermore, the PCO of the weighted Unifrac matrices also showed a higher explained variance than those generated from a Bray Curtis dissimilarity matrix (Table S1).

**Table S1**

|  | | | **Mothur** | **Uparse** | **QIIME**  **closed reference** | **QIIME**  **open reference** |
| --- | --- | --- | --- | --- | --- | --- |
| Successful processing of 80 Gb of MiSeq data | | | No* | Yes | Yes | Yes |
| OTU clustering method | | |  | UPARSE OTU algorithm  ([Edgar, 2013](#_ENREF_3)) | Picks OTUs Greengenes ddb ([McDonald et al., 2012](#_ENREF_5)) | Uclust ([Edgar, 2010](#_ENREF_2)) |
| Total number of paired sequences | | |  | 41,717,147 | 21,736,819 | 39,405,028 |
| Mean number of sequences per sample | | |  | 87,825 | 45,761 | 82,958 |
| Total number of OTUs | | |  | 68,304 | 23,638 | 565,888 |
| Classifier to assign taxonomy | | |  | RDP classifier  ([Wang et al., 2007](#_ENREF_7))  Confidence threshold 51% | Picks OTUs Greengenes ddb | Uclust consensus taxonomy assigner |
| Total number of genera | | |  | 1,982 | 1,782 | 1,903 |
| % variation explained by first 2 PCO axes for water samples ** | Weighted Unifrac | All sequences |  | 38.4 |  | 40.2 |
|  |  | Subsampled 17k sequ. |  | 38.6 |  | 40.6 |
|  | Bray Curtis | All sequences |  | 31.0 |  | 28.7 |
|  |  | Subsampled 17k sequ. |  | 34.2 |  | 31.6 |

* The Mothur pipeline repeatedly crashed at the cluster.split step which assigns sequences to OTUs. This was despite trialling different taxa levels, split methods and mismatch thresholds at the pre-clustering step. This was likely due to a too large distance matrix due to the amount of sequences and potential errors introduced by using paired end MiSeq sequences which did not fully overlap. The median 16s rDNA amplicon length across V4-V5 was 372 bp which compared to 2x 300 bp for the MiSeq v3 chemistry used for rounds 3 to 8 and 2x 250 bp for the v2 chemistry used for the first two rounds.

** Unconstrained principal coordinates ordination (PCO)

**Reference List**

Caporaso, J.G., Kuczynski, J., Stombaugh, J., Bittinger, K., Bushman, F.D., Costello, E.K., et al. (2010). QIIME allows analysis of high-throughput community sequencing data. *Nat Methods* 7(5)**,** 335-336. doi: 10.1038/nmeth.f.303.

Edgar, R.C. (2010). Search and clustering orders of magnitude faster than BLAST. *Bioinformatics* 26(19)**,** 2460-2461. doi: 10.1093/bioinformatics/btq461.

Edgar, R.C. (2013). UPARSE: highly accurate OTU sequences from microbial amplicon reads. *Nature Methods* 10(10)**,** 996-+. doi: 10.1038/Nmeth.2604.

Edgar, R.C., Haas, B.J., Clemente, J.C., Quince, C., and Knight, R. (2011). UCHIME improves sensitivity and speed of chimera detection. *Bioinformatics* 27(16)**,** 2194-2200. doi: 10.1093/bioinformatics/btr381.

McDonald, D., Price, M.N., Goodrich, J., Nawrocki, E.P., DeSantis, T.Z., Probst, A., et al. (2012). An improved Greengenes taxonomy with explicit ranks for ecological and evolutionary analyses of bacteria and archaea. *Isme Journal* 6(3)**,** 610-618. doi: 10.1038/ismej.2011.139.

Schloss, P.D., Westcott, S.L., Ryabin, T., Hall, J.R., Hartmann, M., Hollister, E.B., et al. (2009). Introducing mothur: Open-Source, Platform-Independent, Community-Supported Software for Describing and Comparing Microbial Communities. *Applied and Environmental Microbiology* 75(23)**,** 7537-7541. doi: 10.1128/Aem.01541-09.

Wang, Q., Garrity, G.M., Tiedje, J.M., and Cole, J.R. (2007). Naive Bayesian classifier for rapid assignment of rRNA sequences into the new bacterial taxonomy. *Applied and Environmental Microbiology* 73(16)**,** 5261-5267. doi: 10.1128/Aem.00062-07.
