# Supplementary material for: Spatial and Temporal Microbial Patterns in a Tropical Macrotidal Estuary Subject to Urbanization
Source: Front Microbiol. 2017 Jul 13;8:1313. doi: 10.3389/fmicb.2017.01313 (PMC5507994; doi:10.3389/fmicb.2017.01313)
Supplement: Supplementary file 14 [file Table2.DOCX]

## Table S2 of correct classification rates

Correct classification rates of water samples into their creek of origin for East Arm and Shoal Bay separately. The rates were calculated based on leave-one out allocation in a canonical analysis of principal coordinates (CAP). "n" for total number of samples and "m" for number of PCO axes for the discriminant analysis. Number in brackets are number of samples in category. Misclassifications are listed in order of abundance.

| **Harbour area** | **n,**  **m** | **Creeks**  (No. samples) | **Correct creek classification** | **Misclassifications with** |
| --- | --- | --- | --- | --- |
| East Arm | 139,  23 | Outfall (6) | 67% | Myrmidon |
|  |  | Myrmidon Crk (59) | 42% | Short, Outfall, Ref, Blaydin Pt |
|  |  | Short Crk (30) | 50% | Ref, Myrmidon, Blaydin Pt |
|  |  | Blaydin Pt (15) | 67% | Short, Myrmidon, Ref |
|  |  | Reference Crk (29) | 62% | Short, Blaydin Pt, Myrmidon |
| Shoal Bay | 125,  38 | Outfall (6) | 50% | Buffalo |
|  |  | Buffalo Crk (60) | 80% | Outfall, Micket |
|  |  | Micket Crk (30) | 60% | King |
|  |  | King Crk (29) | 62% | Micket |
