# Supplementary material for: Spatial and Temporal Microbial Patterns in a Tropical Macrotidal Estuary Subject to Urbanization
Source: Front Microbiol. 2017 Jul 13;8:1313. doi: 10.3389/fmicb.2017.01313 (PMC5507994; doi:10.3389/fmicb.2017.01313)
Supplement: Supplementary file 15 [file Table3.DOCX]

# Table S3 Summary of abiotic factors of water samples

## S3 A) East Arm

Median (minimum to maximum) values of abiotic factors of water samples in East Arm in the dry and wet season. Units are ppb or µg/L for all nutrients except not for DOC (mg/L), % for DO, °C for temp, ppt for salinity, NTU for turbidity and µg/L for chlorophyll-a. "OF Myrmidon tributary" effluent outfall into a Myrmidon Creek tributary.

## S3 B) Shoal Bay

Median (minimum to maximum) values of abiotic factors of water samples in Shoal Bay in the dry and wet season. Units are ppb or µg/L for all nutrients except not for DOC (mg/L), % for DO, °C for temp, ppt for salinity, NTU for turbidity and µg/L for chlorophyll-a. "Urban runoff" refers to runoff into the headwaters of Buffalo Creek.
