# Supplementary material for: Spatial and Temporal Microbial Patterns in a Tropical Macrotidal Estuary Subject to Urbanization
Source: Front Microbiol. 2017 Jul 13;8:1313. doi: 10.3389/fmicb.2017.01313 (PMC5507994; doi:10.3389/fmicb.2017.01313)
Supplement: Supplementary file 16 [file Table4.DOCX]

# Table S4 PERMANOVA of sediment microbiota of

## Table S4 A) East Arm

**S4 A) Legend:** Analysis of the microbiota of East Arm sediment samples with **A)** PERMANOVA cross design with fixed factors creeks, years and seasons. Adjusting for multiple testing, only significant associations at P<0.01 are shown. Interactions (IA) between fixed factors are also indicated. The square root estimates of the component of variation are shown in the last column. The residual square root variability was 0.24. **B)** Group comparisons of the weighted Unifrac dissimilarity between creeks. **C)** Correct classification rates of sediment samples into creeks based on CAP (52 PCO axes, leave-one out allocation, n=87 samples). Number in brackets are number of samples in category. Misclassifications are listed in order of abundance.

|  | **Pseudo F**  **(df)** | **P value**  **(>995 perms)** | **Est. comp of variation (sq.root)*** |
| --- | --- | --- | --- |
| Years (2013 vs 2014) | 5.3 (1) | 0.001 | 0.09 |
| Seasons (wet vs dry) | 2.5 (1) | 0.022 | 0.05 |
| IA Years x Seasons | 4.1 (1) | 0.002 | 0.11 |

| **Group comparison** | **Average dissimilarity*** | | | | **T test, P value**  **(>990 perms)** | |
| --- | --- | --- | --- | --- | --- | --- |
|  | **Within group** | | **Btw group** | |  |  |
| Myrmidon Cr | | 0.36 | | vs Short 0.35 | | 1.1, 0.224 |
|  |  |  |  | vs Ref 0.33 | | 1.5, 0.024 |
| Short Cr | | 0.34 | | vs Ref 0.31 | | 1.4, 0.083 |
| Ref Cr | | 0.27 | |  | |  |

| **Sediment samples** | **Correct classification rate** | | **Mis-classifications with** |
| --- | --- | --- | --- |
| Myrmidon Cr (48) | 75% | Ref, Short | |
| Short Cr (19) | 63% | Myrmidon, Ref | |
| Ref Cr (20) | 80% | Myrmidon | |

## Table S4 B) Shoal Bay

**S4 B) Legend**: Analysis of the microbiota of Shoal Bay sediment samples with **A)** PERMANOVA cross design with fixed factors creeks, years and seasons. Adjusting for multiple testing, only significant associations at P<0.01 are shown. Interactions (IA) between fixed factors are also indicated. The square root estimates of the component of variation are shown in the last column. The residual square root variability was 0.29. **B)** Group comparisons of the weighted Unifrac dissimilarity between creeks. **C)** Correct classification rates of sediment samples into creeks based on CAP (79 PCO axes, leave-one out allocation, n=96 samples). Number in brackets are number of samples in category. Misclassifications are listed in order of abundance.

A)

|  | **Pseudo F**  **(df)** | **P value**  **(>995 perms)** | **Est. comp of variation (sq.root)** |
| --- | --- | --- | --- |
| Creeks | 4.0 (2) | 0.001 | 0.10 |
| Years (2013 vs 2014) | 4.8 (1) | 0.001 | 0.09 |
| Seasons (wet vs dry) | 2.8 (1) | 0.003 | 0.06 |
| IA Years x Seasons | 2.7 (1) | 0.006 | 0.08 |

B)

| **Group comparison** | **Average dissimilarity*** | | | | **T test, P value**  **(>995 perms)** | |  |
| --- | --- | --- | --- | --- | --- | --- | --- |
|  | **Within group** | | **Btw group** | |  |  |  |
| Buffalo Cr | | 0.40 | | vs Micket 0.42 | | 2.1, 0.001 | |
|  |  |  |  | vs King 0.45 | | 2.2, 0.001 | |
| Micket Cr | | 0.40 | | vs King 0.44 | | 1.3, 0.044 | |
| King Cr | | 0.46 | |  | |  | |

C)

| **Sediment samples** | **CAP** | **Mis-classifications with** |
| --- | --- | --- |
| Buffalo Cr (54) | 94% | Micket |
| Micket Cr (23) | 91% | Buffalo |
| King Cr (19) | 89% | Micket |
